# Integrative pathway enrichment analysis of multivariate omics data

**DOI:** 10.1038/s41467-019-13983-9

**Published:** 2020-02-05

**Authors:** Marta Paczkowska, Jonathan Barenboim, Nardnisa Sintupisut, Natalie S. Fox, Helen Zhu, Diala Abd-Rabbo, Miles W. Mee, Paul C. Boutros, PCAWG Drivers and Functional Interpretation Working Group, Federico Abascal, Samirkumar B. Amin, Gary D. Bader, Rameen Beroukhim, Johanna Bertl, Keith A. Boroevich, Søren Brunak, Peter J. Campbell, Joana Carlevaro-Fita, Dimple Chakravarty, Calvin Wing Yiu Chan, Ken Chen, Jung Kyoon Choi, Jordi Deu-Pons, Priyanka Dhingra, Klev Diamanti, Lars Feuerbach, J. Lynn Fink, Nuno A. Fonseca, Joan Frigola, Carlo Gambacorti-Passerini, Dale W. Garsed, Mark Gerstein, Gad Getz, Abel Gonzalez-Perez, Qianyun Guo, Ivo G. Gut, David Haan, Mark P. Hamilton, Nicholas J. Haradhvala, Arif O. Harmanci, Mohamed Helmy, Carl Herrmann, Julian M. Hess, Asger Hobolth, Ermin Hodzic, Chen Hong, Henrik Hornshøj, Keren Isaev, Jose M. G. Izarzugaza, Rory Johnson, Todd A. Johnson, Malene Juul, Randi Istrup Juul, Andre Kahles, Abdullah Kahraman, Manolis Kellis, Ekta Khurana, Jaegil Kim, Jong K. Kim, Youngwook Kim, Jan Komorowski, Jan O. Korbel, Sushant Kumar, Andrés Lanzós, Michael S. Lawrence, Donghoon Lee, Kjong-Van Lehmann, Shantao Li, Xiaotong Li, Ziao Lin, Eric Minwei Liu, Lucas Lochovsky, Shaoke Lou, Tobias Madsen, Kathleen Marchal, Iñigo Martincorena, Alexander Martinez-Fundichely, Yosef E. Maruvka, Patrick D. McGillivray, William Meyerson, Ferran Muiños, Loris Mularoni, Hidewaki Nakagawa, Morten Muhlig Nielsen, Keunchil Park, Kiejung Park, Jakob Skou Pedersen, Oriol Pich, Tirso Pons, Sergio Pulido-Tamayo, Benjamin J. Raphael, Iker Reyes-Salazar, Matthew A. Reyna, Esther Rheinbay, Mark A. Rubin, Carlota Rubio-Perez, Radhakrishnan Sabarinathan, S. Cenk Sahinalp, Gordon Saksena, Leonidas Salichos, Chris Sander, Steven E. Schumacher, Mark Shackleton, Ofer Shapira, Ciyue Shen, Raunak Shrestha, Shimin Shuai, Nikos Sidiropoulos, Lina Sieverling, Nasa Sinnott-Armstrong, Lincoln D. Stein, Joshua M. Stuart, David Tamborero, Grace Tiao, Tatsuhiko Tsunoda, Husen M. Umer, Liis Uusküla-Reimand, Alfonso Valencia, Miguel Vazquez, Lieven P. C. Verbeke, Claes Wadelius, Lina Wadi, Jiayin Wang, Jonathan Warrell, Sebastian M. Waszak, Joachim Weischenfeldt, David A. Wheeler, Guanming Wu, Jun Yu, Jing Zhang, Xuanping Zhang, Yan Zhang, Zhongming Zhao, Lihua Zou, Christian von Mering, Jüri Reimand, PCAWG Consortium, Lauri A. Aaltonen, Federico Abascal, Adam Abeshouse, Hiroyuki Aburatani, David J. Adams, Nishant Agrawal, Keun Soo Ahn, Sung-Min Ahn, Hiroshi Aikata, Rehan Akbani, Kadir C. Akdemir, Hikmat Al-Ahmadie, Sultan T. Al-Sedairy, Fatima Al-Shahrour, Malik Alawi, Monique Albert, Kenneth Aldape, Ludmil B. Alexandrov, Adrian Ally, Kathryn Alsop, Eva G. Alvarez, Fernanda Amary, Samirkumar B. Amin, Brice Aminou, Ole Ammerpohl, Matthew J. Anderson, Yeng Ang, Davide Antonello, Pavana Anur, Samuel Aparicio, Elizabeth L. Appelbaum, Yasuhito Arai, Axel Aretz, Koji Arihiro, Shun-ichi Ariizumi, Joshua Armenia, Laurent Arnould, Sylvia Asa, Yassen Assenov, Gurnit Atwal, Sietse Aukema, J. Todd Auman, Miriam R. R. Aure, Philip Awadalla, Marta Aymerich, Gary D. Bader, Adrian Baez-Ortega, Matthew H. Bailey, Peter J. Bailey, Miruna Balasundaram, Saianand Balu, Pratiti Bandopadhayay, Rosamonde E. Banks, Stefano Barbi, Andrew P. Barbour, Jonathan Barenboim, Jill Barnholtz-Sloan, Hugh Barr, Elisabet Barrera, John Bartlett, Javier Bartolome, Claudio Bassi, Oliver F. Bathe, Daniel Baumhoer, Prashant Bavi, Stephen B. Baylin, Wojciech Bazant, Duncan Beardsmore, Timothy A. Beck, Sam Behjati, Andreas Behren, Beifang Niu, Cindy Bell, Sergi Beltran, Christopher Benz, Andrew Berchuck, Anke K. Bergmann, Erik N. Bergstrom, Benjamin P. Berman, Daniel M. Berney, Stephan H. Bernhart, Rameen Beroukhim, Mario Berrios, Samantha Bersani, Johanna Bertl, Miguel Betancourt, Vinayak Bhandari, Shriram G. Bhosle, Andrew V. Biankin, Matthias Bieg, Darell Bigner, Hans Binder, Ewan Birney, Michael Birrer, Nidhan K. Biswas, Bodil Bjerkehagen, Tom Bodenheimer, Lori Boice, Giada Bonizzato, Johann S. De Bono, Arnoud Boot, Moiz S. Bootwalla, Ake Borg, Arndt Borkhardt, Keith A. Boroevich, Ivan Borozan, Christoph Borst, Marcus Bosenberg, Mattia Bosio, Jacqueline Boultwood, Guillaume Bourque, Paul C. Boutros, G. Steven Bova, David T. Bowen, Reanne Bowlby, David D. L. Bowtell, Sandrine Boyault, Rich Boyce, Jeffrey Boyd, Alvis Brazma, Paul Brennan, Daniel S. Brewer, Arie B. Brinkman, Robert G. Bristow, Russell R. Broaddus, Jane E. Brock, Malcolm Brock, Annegien Broeks, Angela N. Brooks, Denise Brooks, Benedikt Brors, Søren Brunak, Timothy J. C. Bruxner, Alicia L. Bruzos, Alex Buchanan, Ivo Buchhalter, Christiane Buchholz, Susan Bullman, Hazel Burke, Birgit Burkhardt, Kathleen H. Burns, John Busanovich, Carlos D. Bustamante, Adam P. Butler, Atul J. Butte, Niall J. Byrne, Anne-Lise Børresen-Dale, Samantha J. Caesar-Johnson, Andy Cafferkey, Declan Cahill, Claudia Calabrese, Carlos Caldas, Fabien Calvo, Niedzica Camacho, Peter J. Campbell, Elias Campo, Cinzia Cantù, Shaolong Cao, Thomas E. Carey, Joana Carlevaro-Fita, Rebecca Carlsen, Ivana Cataldo, Mario Cazzola, Jonathan Cebon, Robert Cerfolio, Dianne E. Chadwick, Dimple Chakravarty, Don Chalmers, Calvin Wing Yiu Chan, Kin Chan, Michelle Chan-Seng-Yue, Vishal S. Chandan, David K. Chang, Stephen J. Chanock, Lorraine A. Chantrill, Aurélien Chateigner, Nilanjan Chatterjee, Kazuaki Chayama, Hsiao-Wei Chen, Jieming Chen, Ken Chen, Yiwen Chen, Zhaohong Chen, Andrew D. Cherniack, Jeremy Chien, Yoke-Eng Chiew, Suet-Feung Chin, Juok Cho, Sunghoon Cho, Jung Kyoon Choi, Wan Choi, Christine Chomienne, Zechen Chong, Su Pin Choo, Angela Chou, Angelika N. Christ, Elizabeth L. Christie, Eric Chuah, Carrie Cibulskis, Kristian Cibulskis, Sara Cingarlini, Peter Clapham, Alexander Claviez, Sean Cleary, Nicole Cloonan, Marek Cmero, Colin C. Collins, Ashton A. Connor, Susanna L. Cooke, Colin S. Cooper, Leslie Cope, Vincenzo Corbo, Matthew G. Cordes, Stephen M. Cordner, Isidro Cortés-Ciriano, Kyle Covington, Prue A. Cowin, Brian Craft, David Craft, Chad J. Creighton, Yupeng Cun, Erin Curley, Ioana Cutcutache, Karolina Czajka, Bogdan Czerniak, Rebecca A. Dagg, Ludmila Danilova, Maria Vittoria Davi, Natalie R. Davidson, Helen Davies, Ian J. Davis, Brandi N. Davis-Dusenbery, Kevin J. Dawson, Francisco M. De La Vega, Ricardo De Paoli-Iseppi, Timothy Defreitas, Angelo P. Dei Tos, Olivier Delaneau, John A. Demchok, Jonas Demeulemeester, German M. Demidov, Deniz Demircioğlu, Nening M. Dennis, Robert E. Denroche, Stefan C. Dentro, Nikita Desai, Vikram Deshpande, Amit G. Deshwar, Christine Desmedt, Jordi Deu-Pons, Noreen Dhalla, Neesha C. Dhani, Priyanka Dhingra, Rajiv Dhir, Anthony DiBiase, Klev Diamanti, Li Ding, Shuai Ding, Huy Q. Dinh, Luc Dirix, HarshaVardhan Doddapaneni, Nilgun Donmez, Michelle T. Dow, Ronny Drapkin, Oliver Drechsel, Ruben M. Drews, Serge Serge, Tim Dudderidge, Ana Dueso-Barroso, Andrew J. Dunford, Michael Dunn, Lewis Jonathan Dursi, Fraser R. Duthie, Ken Dutton-Regester, Jenna Eagles, Douglas F. Easton, Stuart Edmonds, Paul A. Edwards, Sandra E. Edwards, Rosalind A. Eeles, Anna Ehinger, Juergen Eils, Roland Eils, Adel El-Naggar, Matthew Eldridge, Kyle Ellrott, Serap Erkek, Georgia Escaramis, Shadrielle M. G. Espiritu, Xavier Estivill, Dariush Etemadmoghadam, Jorunn E. Eyfjord, Bishoy M. Faltas, Daiming Fan, Yu Fan, William C. Faquin, Claudiu Farcas, Matteo Fassan, Aquila Fatima, Francesco Favero, Nodirjon Fayzullaev, Ina Felau, Sian Fereday, Martin L. Ferguson, Vincent Ferretti, Lars Feuerbach, Matthew A. Field, J. Lynn Fink, Gaetano Finocchiaro, Cyril Fisher, Matthew W. Fittall, Anna Fitzgerald, Rebecca C. Fitzgerald, Adrienne M. Flanagan, Neil E. Fleshner, Paul Flicek, John A. Foekens, Kwun M. Fong, Nuno A. Fonseca, Christopher S. Foster, Natalie S. Fox, Michael Fraser, Scott Frazer, Milana Frenkel-Morgenstern, William Friedman, Joan Frigola, Catrina C. Fronick, Akihiro Fujimoto, Masashi Fujita, Masashi Fukayama, Lucinda A. Fulton, Robert S. Fulton, Mayuko Furuta, P. Andrew Futreal, Anja Füllgrabe, Stacey B. Gabriel, Steven Gallinger, Carlo Gambacorti-Passerini, Jianjiong Gao, Shengjie Gao, Levi Garraway, Øystein Garred, Erik Garrison, Dale W. Garsed, Nils Gehlenborg, Josep L. L. Gelpi, Joshy George, Daniela S. Gerhard, Clarissa Gerhauser, Jeffrey E. Gershenwald, Mark Gerstein, Moritz Gerstung, Gad Getz, Mohammed Ghori, Ronald Ghossein, Nasra H. Giama, Richard A. Gibbs, Bob Gibson, Anthony J. Gill, Pelvender Gill, Dilip D. Giri, Dominik Glodzik, Vincent J. Gnanapragasam, Maria Elisabeth Goebler, Mary J. Goldman, Carmen Gomez, Santiago Gonzalez, Abel Gonzalez-Perez, Dmitry A. Gordenin, James Gossage, Kunihito Gotoh, Ramaswamy Govindan, Dorthe Grabau, Janet S. Graham, Robert C. Grant, Anthony R. Green, Eric Green, Liliana Greger, Nicola Grehan, Sonia Grimaldi, Sean M. Grimmond, Robert L. Grossman, Adam Grundhoff, Gunes Gundem, Qianyun Guo, Manaswi Gupta, Shailja Gupta, Ivo G. Gut, Marta Gut, Jonathan Göke, Gavin Ha, Andrea Haake, David Haan, Siegfried Haas, Kerstin Haase, James E. Haber, Nina Habermann, Faraz Hach, Syed Haider, Natsuko Hama, Freddie C. Hamdy, Anne Hamilton, Mark P. Hamilton, Leng Han, George B. Hanna, Martin Hansmann, Nicholas J. Haradhvala, Olivier Harismendy, Ivon Harliwong, Arif O. Harmanci, Eoghan Harrington, Takanori Hasegawa, David Haussler, Steve Hawkins, Shinya Hayami, Shuto Hayashi, D. Neil Hayes, Stephen J. Hayes, Nicholas K. Hayward, Steven Hazell, Yao He, Allison P. Heath, Simon C. Heath, David Hedley, Apurva M. Hegde, David I. Heiman, Michael C. Heinold, Zachary Heins, Lawrence E. Heisler, Eva Hellstrom-Lindberg, Mohamed Helmy, Seong Gu Heo, Austin J. Hepperla, José María Heredia-Genestar, Carl Herrmann, Peter Hersey, Julian M. Hess, Holmfridur Hilmarsdottir, Jonathan Hinton, Satoshi Hirano, Nobuyoshi Hiraoka, Katherine A. Hoadley, Asger Hobolth, Ermin Hodzic, Jessica I. Hoell, Steve Hoffmann, Oliver Hofmann, Andrea Holbrook, Aliaksei Z. Holik, Michael A. Hollingsworth, Oliver Holmes, Robert A. Holt, Chen Hong, Eun Pyo Hong, Jongwhi H. Hong, Gerrit K. Hooijer, Henrik Hornshøj, Fumie Hosoda, Yong Hou, Volker Hovestadt, William Howat, Alan P. Hoyle, Ralph H. Hruban, Jianhong Hu, Taobo Hu, Xing Hua, Kuan-lin Huang, Mei Huang, Mi Ni Huang, Vincent Huang, Yi Huang, Wolfgang Huber, Thomas J. Hudson, Michael Hummel, Jillian A. Hung, David Huntsman, Ted R. Hupp, Jason Huse, Matthew R. Huska, Barbara Hutter, Carolyn M. Hutter, Daniel Hübschmann, Christine A. Iacobuzio-Donahue, Charles David Imbusch, Marcin Imielinski, Seiya Imoto, William B. Isaacs, Keren Isaev, Shumpei Ishikawa, Murat Iskar, S. M. Ashiqul Islam, Michael Ittmann, Sinisa Ivkovic, Jose M. G. Izarzugaza, Jocelyne Jacquemier, Valerie Jakrot, Nigel B. Jamieson, Gun Ho Jang, Se Jin Jang, Joy C. Jayaseelan, Reyka Jayasinghe, Stuart R. Jefferys, Karine Jegalian, Jennifer L. Jennings, Seung-Hyup Jeon, Lara Jerman, Yuan Ji, Wei Jiao, Peter A. Johansson, Amber L. Johns, Jeremy Johns, Rory Johnson, Todd A. Johnson, Clemency Jolly, Yann Joly, Jon G. Jonasson, Corbin D. Jones, David R. Jones, David T. W. Jones, Nic Jones, Steven J. M. Jones, Jos Jonkers, Young Seok Ju, Hartmut Juhl, Jongsun Jung, Malene Juul, Randi Istrup Juul, Sissel Juul, Natalie Jäger, Rolf Kabbe, Andre Kahles, Abdullah Kahraman, Vera B. Kaiser, Hojabr Kakavand, Sangeetha Kalimuthu, Christof von Kalle, Koo Jeong Kang, Katalin Karaszi, Beth Karlan, Rosa Karlić, Dennis Karsch, Katayoon Kasaian, Karin S. Kassahn, Hitoshi Katai, Mamoru Kato, Hiroto Katoh, Yoshiiku Kawakami, Jonathan D. Kay, Stephen H. Kazakoff, Marat D. Kazanov, Maria Keays, Electron Kebebew, Richard F. Kefford, Manolis Kellis, James G. Kench, Catherine J. Kennedy, Jules N. A. Kerssemakers, David Khoo, Vincent Khoo, Narong Khuntikeo, Ekta Khurana, Helena Kilpinen, Hark Kyun Kim, Hyung-Lae Kim, Hyung-Yong Kim, Hyunghwan Kim, Jaegil Kim, Jihoon Kim, Jong K. Kim, Youngwook Kim, Tari A. King, Wolfram Klapper, Kortine Kleinheinz, Leszek J. Klimczak, Stian Knappskog, Michael Kneba, Bartha M. Knoppers, Youngil Koh, Jan Komorowski, Daisuke Komura, Mitsuhiro Komura, Gu Kong, Marcel Kool, Jan O. Korbel, Viktoriya Korchina, Andrey Korshunov, Michael Koscher, Roelof Koster, Zsofia Kote-Jarai, Antonios Koures, Milena Kovacevic, Barbara Kremeyer, Helene Kretzmer, Markus Kreuz, Savitri Krishnamurthy, Dieter Kube, Kiran Kumar, Pardeep Kumar, Sushant Kumar, Yogesh Kumar, Ritika Kundra, Kirsten Kübler, Ralf Küppers, Jesper Lagergren, Phillip H. Lai, Peter W. Laird, Sunil R. Lakhani, Christopher M. Lalansingh, Emilie Lalonde, Fabien C. Lamaze, Adam Lambert, Eric Lander, Pablo Landgraf, Luca Landoni, Anita Langerød, Andrés Lanzós, Denis Larsimont, Erik Larsson, Mark Lathrop, Loretta M. S. Lau, Chris Lawerenz, Rita T. Lawlor, Michael S. Lawrence, Alexander J. Lazar, Ana Mijalkovic Lazic, Xuan Le, Darlene Lee, Donghoon Lee, Eunjung Alice Lee, Hee Jin Lee, Jake June-Koo Lee, Jeong-Yeon Lee, Juhee Lee, Ming Ta Michael Lee, Henry Lee-Six, Kjong-Van Lehmann, Hans Lehrach, Dido Lenze, Conrad R. Leonard, Daniel A. Leongamornlert, Ignaty Leshchiner, Louis Letourneau, Ivica Letunic, Douglas A. Levine, Lora Lewis, Tim Ley, Chang Li, Constance H. Li, Haiyan Irene Li, Jun Li, Lin Li, Shantao Li, Siliang Li, Xiaobo Li, Xiaotong Li, Xinyue Li, Yilong Li, Han Liang, Sheng-Ben Liang, Peter Lichter, Pei Lin, Ziao Lin, W. M. Linehan, Ole Christian Lingjærde, Dongbing Liu, Eric Minwei Liu, Fei-Fei Fei Liu, Fenglin Liu, Jia Liu, Xingmin Liu, Julie Livingstone, Dimitri Livitz, Naomi Livni, Lucas Lochovsky, Markus Loeffler, Georgina V. Long, Armando Lopez-Guillermo, Shaoke Lou, David N. Louis, Laurence B. Lovat, Yiling Lu, Yong-Jie Lu, Youyong Lu, Claudio Luchini, Ilinca Lungu, Xuemei Luo, Hayley J. Luxton, Andy G. Lynch, Lisa Lype, Cristina López, Carlos López-Otín, Eric Z. Ma, Yussanne Ma, Gaetan MacGrogan, Shona MacRae, Geoff Macintyre, Tobias Madsen, Kazuhiro Maejima, Andrea Mafficini, Dennis T. Maglinte, Arindam Maitra, Partha P. Majumder, Luca Malcovati, Salem Malikic, Giuseppe Malleo, Graham J. Mann, Luisa Mantovani-Löffler, Kathleen Marchal, Giovanni Marchegiani, Elaine R. Mardis, Adam A. Margolin, Maximillian G. Marin, Florian Markowetz, Julia Markowski, Jeffrey Marks, Tomas Marques-Bonet, Marco A. Marra, Luke Marsden, John W. M. Martens, Sancha Martin, Jose I. Martin-Subero, Iñigo Martincorena, Alexander Martinez-Fundichely, Yosef E. Maruvka, R. Jay Mashl, Charlie E. Massie, Thomas J. Matthew, Lucy Matthews, Erik Mayer, Simon Mayes, Michael Mayo, Faridah Mbabaali, Karen McCune, Ultan McDermott, Patrick D. McGillivray, Michael D. McLellan, John D. McPherson, John R. McPherson, Treasa A. McPherson, Samuel R. Meier, Alice Meng, Shaowu Meng, Andrew Menzies, Neil D. Merrett, Sue Merson, Matthew Meyerson, William Meyerson, Piotr A. Mieczkowski, George L. Mihaiescu, Sanja Mijalkovic, Tom Mikkelsen, Michele Milella, Linda Mileshkin, Christopher A. Miller, David K. Miller, Jessica K. Miller, Gordon B. Mills, Ana Milovanovic, Sarah Minner, Marco Miotto, Gisela Mir Arnau, Lisa Mirabello, Chris Mitchell, Thomas J. Mitchell, Satoru Miyano, Naoki Miyoshi, Shinichi Mizuno, Fruzsina Molnár-Gábor, Malcolm J. Moore, Richard A. Moore, Sandro Morganella, Quaid D. Morris, Carl Morrison, Lisle E. Mose, Catherine D. Moser, Ferran Muiños, Loris Mularoni, Andrew J. Mungall, Karen Mungall, Elizabeth A. Musgrove, Ville Mustonen, David Mutch, Francesc Muyas, Donna M. Muzny, Alfonso Muñoz, Jerome Myers, Ola Myklebost, Peter Möller, Genta Nagae, Adnan M. Nagrial, Hardeep K. Nahal-Bose, Hitoshi Nakagama, Hidewaki Nakagawa, Hiromi Nakamura, Toru Nakamura, Kaoru Nakano, Tannistha Nandi, Jyoti Nangalia, Mia Nastic, Arcadi Navarro, Fabio C. P. Navarro, David E. Neal, Gerd Nettekoven, Felicity Newell, Steven J. Newhouse, Yulia Newton, Alvin Wei Tian Ng, Anthony Ng, Jonathan Nicholson, David Nicol, Yongzhan Nie, G. Petur Nielsen, Morten Muhlig Nielsen, Serena Nik-Zainal, Michael S. Noble, Katia Nones, Paul A. Northcott, Faiyaz Notta, Brian D. O’Connor, Peter O’Donnell, Maria O’Donovan, Sarah O’Meara, Brian Patrick O’Neill, J. Robert O’Neill, David Ocana, Angelica Ochoa, Layla Oesper, Christopher Ogden, Hideki Ohdan, Kazuhiro Ohi, Lucila Ohno-Machado, Karin A. Oien, Akinyemi I. Ojesina, Hidenori Ojima, Takuji Okusaka, Larsson Omberg, Choon Kiat Ong, Stephan Ossowski, German Ott, B. F. Francis Ouellette, Christine P’ng, Marta Paczkowska, Salvatore Paiella, Chawalit Pairojkul, Marina Pajic, Qiang Pan-Hammarström, Elli Papaemmanuil, Irene Papatheodorou, Nagarajan Paramasivam, Ji Wan Park, Joong-Won Park, Keunchil Park, Kiejung Park, Peter J. Park, Joel S. Parker, Simon L. Parsons, Harvey Pass, Danielle Pasternack, Alessandro Pastore, Ann-Marie Patch, Iris Pauporté, Antonio Pea, John V. Pearson, Chandra Sekhar Pedamallu, Jakob Skou Pedersen, Paolo Pederzoli, Martin Peifer, Nathan A. Pennell, Charles M. Perou, Marc D. Perry, Gloria M. Petersen, Myron Peto, Nicholas Petrelli, Robert Petryszak, Stefan M. Pfister, Mark Phillips, Oriol Pich, Hilda A. Pickett, Todd D. Pihl, Nischalan Pillay, Sarah Pinder, Mark Pinese, Andreia V. Pinho, Esa Pitkänen, Xavier Pivot, Elena Piñeiro-Yáñez, Laura Planko, Christoph Plass, Paz Polak, Tirso Pons, Irinel Popescu, Olga Potapova, Aparna Prasad, Shaun R. Preston, Manuel Prinz, Antonia L. Pritchard, Stephenie D. Prokopec, Elena Provenzano, Xose S. Puente, Sonia Puig, Montserrat Puiggròs, Sergio Pulido-Tamayo, Gulietta M. Pupo, Colin A. Purdie, Michael C. Quinn, Raquel Rabionet, Janet S. Rader, Bernhard Radlwimmer, Petar Radovic, Benjamin Raeder, Keiran M. Raine, Manasa Ramakrishna, Kamna Ramakrishnan, Suresh Ramalingam, Benjamin J. Raphael, W. Kimryn Rathmell, Tobias Rausch, Guido Reifenberger, Jüri Reimand, Jorge Reis-Filho, Victor Reuter, Iker Reyes-Salazar, Matthew A. Reyna, Sheila M. Reynolds, Esther Rheinbay, Yasser Riazalhosseini, Andrea L. Richardson, Julia Richter, Matthew Ringel, Markus Ringnér, Yasushi Rino, Karsten Rippe, Jeffrey Roach, Lewis R. Roberts, Nicola D. Roberts, Steven A. Roberts, A. Gordon Robertson, Alan J. Robertson, Javier Bartolomé Rodriguez, Bernardo Rodriguez-Martin, F. Germán Rodríguez-González, Michael H. A. Roehrl, Marius Rohde, Hirofumi Rokutan, Gilles Romieu, Ilse Rooman, Tom Roques, Daniel Rosebrock, Mara Rosenberg, Philip C. Rosenstiel, Andreas Rosenwald, Edward W. Rowe, Romina Royo, Steven G. Rozen, Yulia Rubanova, Mark A. Rubin, Carlota Rubio-Perez, Vasilisa A. Rudneva, Borislav C. Rusev, Andrea Ruzzenente, Gunnar Rätsch, Radhakrishnan Sabarinathan, Veronica Y. Sabelnykova, Sara Sadeghi, S. Cenk Sahinalp, Natalie Saini, Mihoko Saito-Adachi, Gordon Saksena, Adriana Salcedo, Roberto Salgado, Leonidas Salichos, Richard Sallari, Charles Saller, Roberto Salvia, Michelle Sam, Jaswinder S. Samra, Francisco Sanchez-Vega, Chris Sander, Grant Sanders, Rajiv Sarin, Iman Sarrafi, Aya Sasaki-Oku, Torill Sauer, Guido Sauter, Robyn P. M. Saw, Maria Scardoni, Christopher J. Scarlett, Aldo Scarpa, Ghislaine Scelo, Dirk Schadendorf, Jacqueline E. Schein, Markus B. Schilhabel, Matthias Schlesner, Thorsten Schlomm, Heather K. Schmidt, Sarah-Jane Schramm, Stefan Schreiber, Nikolaus Schultz, Steven E. Schumacher, Roland F. Schwarz, Richard A. Scolyer, David Scott, Ralph Scully, Raja Seethala, Ayellet V. Segre, Iris Selander, Colin A. Semple, Yasin Senbabaoglu, Subhajit Sengupta, Elisabetta Sereni, Stefano Serra, Dennis C. Sgroi, Mark Shackleton, Nimish C. Shah, Sagedeh Shahabi, Catherine A. Shang, Ping Shang, Ofer Shapira, Troy Shelton, Ciyue Shen, Hui Shen, Rebecca Shepherd, Ruian Shi, Yan Shi, Yu-Jia Shiah, Tatsuhiro Shibata, Juliann Shih, Eigo Shimizu, Kiyo Shimizu, Seung Jun Shin, Yuichi Shiraishi, Tal Shmaya, Ilya Shmulevich, Solomon I. Shorser, Charles Short, Raunak Shrestha, Suyash S. Shringarpure, Craig Shriver, Shimin Shuai, Nikos Sidiropoulos, Reiner Siebert, Anieta M. Sieuwerts, Lina Sieverling, Sabina Signoretti, Katarzyna O. Sikora, Michele Simbolo, Ronald Simon, Janae V. Simons, Jared T. Simpson, Peter T. Simpson, Samuel Singer, Nasa Sinnott-Armstrong, Payal Sipahimalani, Tara J. Skelly, Marcel Smid, Jaclyn Smith, Karen Smith-McCune, Nicholas D. Socci, Heidi J. Sofia, Matthew G. Soloway, Lei Song, Anil K. Sood, Sharmila Sothi, Christos Sotiriou, Cameron M. Soulette, Paul N. Span, Paul T. Spellman, Nicola Sperandio, Andrew J. Spillane, Oliver Spiro, Jonathan Spring, Johan Staaf, Peter F. Stadler, Peter Staib, Stefan G. Stark, Lucy Stebbings, Ólafur Andri Stefánsson, Oliver Stegle, Lincoln D. Stein, Alasdair Stenhouse, Chip Stewart, Stephan Stilgenbauer, Miranda D. Stobbe, Michael R. Stratton, Jonathan R. Stretch, Adam J. Struck, Joshua M. Stuart, Henk G. Stunnenberg, Hong Su, Xiaoping Su, Ren X. Sun, Stephanie Sungalee, Hana Susak, Akihiro Suzuki, Fred Sweep, Monika Szczepanowski, Holger Sültmann, Takashi Yugawa, Angela Tam, David Tamborero, Benita Kiat Tee Tan, Donghui Tan, Patrick Tan, Hiroko Tanaka, Hirokazu Taniguchi, Tomas J. Tanskanen, Maxime Tarabichi, Roy Tarnuzzer, Patrick Tarpey, Morgan L. Taschuk, Kenji Tatsuno, Simon Tavaré, Darrin F. Taylor, Amaro Taylor-Weiner, Jon W. Teague, Bin Tean Teh, Varsha Tembe, Javier Temes, Kevin Thai, Sarah P. Thayer, Nina Thiessen, Gilles Thomas, Sarah Thomas, Alan Thompson, Alastair M. Thompson, John F. F. Thompson, R. Houston Thompson, Heather Thorne, Leigh B. Thorne, Adrian Thorogood, Grace Tiao, Nebojsa Tijanic, Lee E. Timms, Roberto Tirabosco, Marta Tojo, Stefania Tommasi, Christopher W. Toon, Umut H. Toprak, David Torrents, Giampaolo Tortora, Jörg Tost, Yasushi Totoki, David Townend, Nadia Traficante, Isabelle Treilleux, Jean-Rémi Trotta, Lorenz H. P. Trümper, Ming Tsao, Tatsuhiko Tsunoda, Jose M. C. Tubio, Olga Tucker, Richard Turkington, Daniel J. Turner, Andrew Tutt, Masaki Ueno, Naoto T. Ueno, Christopher Umbricht, Husen M. Umer, Timothy J. Underwood, Lara Urban, Tomoko Urushidate, Tetsuo Ushiku, Liis Uusküla-Reimand, Alfonso Valencia, David J. Van Den Berg, Steven Van Laere, Peter Van Loo, Erwin G. Van Meir, Gert G. Van den Eynden, Theodorus Van der Kwast, Naveen Vasudev, Miguel Vazquez, Ravikiran Vedururu, Umadevi Veluvolu, Shankar Vembu, Lieven P. C. Verbeke, Peter Vermeulen, Clare Verrill, Alain Viari, David Vicente, Caterina Vicentini, K. VijayRaghavan, Juris Viksna, Ricardo E. Vilain, Izar Villasante, Anne Vincent-Salomon, Tapio Visakorpi, Douglas Voet, Paresh Vyas, Ignacio Vázquez-García, Nick M. Waddell, Nicola Waddell, Claes Wadelius, Lina Wadi, Rabea Wagener, Jeremiah A. Wala, Jian Wang, Jiayin Wang, Linghua Wang, Qi Wang, Wenyi Wang, Yumeng Wang, Zhining Wang, Paul M. Waring, Hans-Jörg Warnatz, Jonathan Warrell, Anne Y. Warren, Sebastian M. Waszak, David C. Wedge, Dieter Weichenhan, Paul Weinberger, John N. Weinstein, Joachim Weischenfeldt, Daniel J. Weisenberger, Ian Welch, Michael C. Wendl, Johannes Werner, Justin P. Whalley, David A. Wheeler, Hayley C. Whitaker, Dennis Wigle, Matthew D. Wilkerson, Ashley Williams, James S. Wilmott, Gavin W. Wilson, Julie M. Wilson, Richard K. Wilson, Boris Winterhoff, Jeffrey A. Wintersinger, Maciej Wiznerowicz, Stephan Wolf, Bernice H. Wong, Tina Wong, Winghing Wong, Youngchoon Woo, Scott Wood, Bradly G. Wouters, Adam J. Wright, Derek W. Wright, Mark H. Wright, Chin-Lee Wu, Dai-Ying Wu, Guanming Wu, Jianmin Wu, Kui Wu, Yang Wu, Zhenggang Wu, Liu Xi, Tian Xia, Qian Xiang, Xiao Xiao, Rui Xing, Heng Xiong, Qinying Xu, Yanxun Xu, Hong Xue, Shinichi Yachida, Sergei Yakneen, Rui Yamaguchi, Takafumi N. Yamaguchi, Masakazu Yamamoto, Shogo Yamamoto, Hiroki Yamaue, Fan Yang, Huanming Yang, Jean Y. Yang, Liming Yang, Lixing Yang, Shanlin Yang, Tsun-Po Yang, Yang Yang, Xiaotong Yao, Marie-Laure Yaspo, Lucy Yates, Christina Yau, Chen Ye, Kai Ye, Venkata D. Yellapantula, Christopher J. Yoon, Sung-Soo Yoon, Fouad Yousif, Jun Yu, Kaixian Yu, Willie Yu, Yingyan Yu, Ke Yuan, Yuan Yuan, Denis Yuen, Christina K. Yung, Olga Zaikova, Jorge Zamora, Marc Zapatka, Jean C. Zenklusen, Thorsten Zenz, Nikolajs Zeps, Cheng-Zhong Zhang, Fan Zhang, Hailei Zhang, Hongwei Zhang, Hongxin Zhang, Jiashan Zhang, Jing Zhang, Junjun Zhang, Xiuqing Zhang, Xuanping Zhang, Yan Zhang, Zemin Zhang, Zhongming Zhao, Liangtao Zheng, Xiuqing Zheng, Wanding Zhou, Yong Zhou, Bin Zhu, Hongtu Zhu, Jingchun Zhu, Shida Zhu, Lihua Zou, Xueqing Zou, Anna deFazio, Nicholas van As, Carolien H. M. van Deurzen, Marc J. van de Vijver, L. van’t Veer, Christian von Mering

**Affiliations:** 1 Computational Biology Program, Ontario Institute for Cancer Research, 661 University Ave Suite 510, Toronto, ON M5G 0A3 Canada; 2 Department of Medical Biophysics, University of Toronto, 101 College Street Suite 15-701, Toronto, ON M5G 1L7 Canada; 3 Department of Pharmacology & Toxicology, University of Toronto, 1 King’s College Circle Room 4207, Toronto, ON M5S 1A8 Canada; 4 Department of Human Genetics, University of California Los Angeles, 10833 Le Conte Avenue, Los Angeles, CA 90095 USA; 5 Department of Urology, University of California Los Angeles, 200 Medical Plaza Driveway #140, Los Angeles, CA 90024 USA; 6 Institute of Precision Health, University of California Los Angeles, 10833 Le Conte Avenue, Los Angeles, CA 90024 USA; 7 Broad Stem Cell Research Centre, University of California Los Angeles, 615 Charles E Young Drive S, Los Angeles, CA 90095 USA; 8 Jonsson Comprehensive Cancer Centre, University of California Los Angeles, 10833 Le Conte Avenue, Los Angeles, CA 90024 USA; 9 Wellcome Sanger Institute, Wellcome Genome Campus, Hinxton, Cambridge CB10 1SA UK; 10 Department of Genomic Medicine, The University of Texas MD Anderson Cancer Center, Houston, TX 77030 USA; 11 Quantitative & Computational Biosciences Graduate Program, Baylor College of Medicine, Houston, TX 77030 USA; 12 Department of Molecular Genetics, University of Toronto, Toronto, ON M5S 1A8 Canada; 13 Broad Institute of MIT and Harvard, Cambridge, MA 02142 USA; 14 Department of Medical Oncology, Dana-Farber Cancer Institute, Boston, MA 02115 USA; 15 Harvard Medical School, Boston, MA 02115 USA; 16 Department of Mathematics, Aarhus University, 8000 Aarhus, Denmark; 17 Department of Molecular Medicine (MOMA), Aarhus University Hospital, 8200 Aarhus N, Denmark; 18 Laboratory for Medical Science Mathematics, RIKEN Center for Integrative Medical Sciences, Yokohama, Kanagawa 230-0045 Japan; 19 RIKEN Center for Integrative Medical Sciences, Yokohama, Kanagawa 230-0045 Japan; 20 Technical University of Denmark, 2800 Lyngby, Denmark; 21 University of Copenhagen, 2200 Copenhagen, Denmark; 22 Department of Haematology, University of Cambridge, Cambridge, CB2 2XY UK; 23 Department for BioMedical Research, University of Bern, 3008 Bern, Switzerland; 24 Department of Medical Oncology, Inselspital, University Hospital and University of Bern, 3010 Bern, Switzerland; 25 Graduate School for Cellular and Biomedical Sciences, University of Bern, 3012 Bern, Switzerland; 26 Department of Genitourinary Medical Oncology—Research, Division of Cancer Medicine, The University of Texas MD Anderson Cancer Center, Houston, TX 77030 USA; 27 Division of Theoretical Bioinformatics, German Cancer Research Center (DKFZ), 69120 Heidelberg, Germany; 28 Faculty of Biosciences, Heidelberg University, 69120 Heidelberg, Germany; 29 University of Texas MD Anderson Cancer Center, Houston, TX 77030 USA; 30 Korea Advanced Institute of Science and Technology, Daejeon, 34141 South Korea; 31 Institute for Research in Biomedicine (IRB Barcelona), The Barcelona Institute of Science and Technology, 8003 Barcelona, Spain; 32 Research Program on Biomedical Informatics, Universitat Pompeu Fabra, 08002 Barcelona, Spain; 33 Department of Physiology and Biophysics, Weill Cornell Medicine, New York, NY 10065 USA; 34 Institute for Computational Biomedicine, Weill Cornell Medicine, New York, NY 10021 USA; 35 Science for Life Laboratory, Department of Cell and Molecular Biology, Uppsala University, SE-75124 Uppsala, Sweden; 36 Division of Applied Bioinformatics, German Cancer Research Center (DKFZ), 69120 Heidelberg, Germany; 37 Barcelona Supercomputing Center, 08034 Barcelona, Spain; 38 Queensland Centre for Medical Genomics, Institute for Molecular Bioscience, The University of Queensland, St Lucia, QLD 4072 Australia; 39 CIBIO/InBIO—Research Center in Biodiversity and Genetic Resources, Universidade do Porto, 4485-601 Vairão, Portugal; 40 European Bioinformatics Institute (EMBL-EBI), Wellcome Genome Campus, Hinxton, Cambridge CB10 1SD UK; 41 University of Milano Bicocca, 20052 Monza, Italy; 42 Peter MacCallum Cancer Centre, Melbourne, VIC 3000 Australia; 43 Sir Peter MacCallum Department of Oncology, The University of Melbourne, Melbourne, VIC 3052 Australia; 44 Department of Computer Science, Princeton University, Princeton, NJ 08540 USA; 45 Department of Computer Science, Yale University, New Haven, CT 06520 USA; 46 Department of Molecular Biophysics and Biochemistry, Yale University, New Haven, CT 06520 USA; 47 Program in Computational Biology and Bioinformatics, Yale University, New Haven, CT 06520 USA; 48 Center for Cancer Research, Massachusetts General Hospital, Boston, MA 02129 USA; 49 Department of Pathology, Massachusetts General Hospital, Boston, MA 02115 USA; 50 Institut Hospital del Mar d’Investigacions Mèdiques (IMIM), 08003 Barcelona, Spain; 51 Institute for Research in Biomedicine (IRB Barcelona), The Barcelona Institute of Science and Technology, Barcelona, Spain; 52 Bioinformatics Research Centre (BiRC), Aarhus University, 8000 Aarhus, Denmark; 53 CNAG-CRG, Centre for Genomic Regulation (CRG), Barcelona Institute of Science and Technology (BIST), 08028 Barcelona, Spain; 54 Universitat Pompeu Fabra (UPF), 08003 Barcelona, Spain; 55 Biomolecular Engineering Department, University of California, Santa Cruz, Santa Cruz, CA 95064 USA; 56 Department of Internal Medicine, Stanford University, Stanford, CA 94305 USA; 57 Massachusetts General Hospital, Boston, MA 02114 USA; 58 Center for Precision Health, School of Biomedical Informatics, University of Texas Health Science Center, Houston, TX 77030 USA; 59 The Donnelly Centre, University of Toronto, Toronto, ON M5S 3E1 Canada; 60 Health Data Science Unit, University Clinics, 69120 Heidelberg, Germany; 61 Institute of Pharmacy and Molecular Biotechnology and BioQuant, Heidelberg University, 69120 Heidelberg, Germany; 62 Massachusetts General Hospital Center for Cancer Research, Charlestown, MA 02129 USA; 63 Simon Fraser University, Burnaby, BC V5A 1S6 Canada; 64 Department for Biomedical Research, University of Bern, 3008 Bern, Switzerland; 65 Computational Biology Center, Memorial Sloan Kettering Cancer Center, New York, NY 10065 USA; 66 Department of Biology, ETH Zurich, Wolfgang-Pauli-Strasse 27, 8093 Zürich, Switzerland; 67 Department of Computer Science, ETH Zurich, 8092 Zurich, Switzerland; 68 SIB Swiss Institute of Bioinformatics, 1015 Lausanne, Switzerland; 69 University Hospital Zurich, 8091 Zurich, Switzerland; 70 Clinical Bioinformatics, Swiss Institute of Bioinformatics, 1202 Geneva, Switzerland; 71 Institute for Pathology and Molecular Pathology, University Hospital Zurich, 8091 Zurich, Switzerland; 72 Institute of Molecular Life Sciences, University of Zurich, 8057 Zurich, Switzerland; 73 MIT Computer Science and Artificial Intelligence Laboratory, Massachusetts Institute of Technology, Cambridge, MA 02139 USA; 74 Englander Institute for Precision Medicine, Weill Cornell Medicine, New York, NY 10065 USA; 75 Meyer Cancer Center, Weill Cornell Medicine, New York, NY 10065 USA; 76 Research Core Center, National Cancer Centre Korea, Goyang-si, 410-769 South Korea; 77 Department of Health Sciences and Technology, Sungkyunkwan University School of Medicine, Seoul, 06351 South Korea; 78 Samsung Genome Institute, Samsung Medical Center, Seoul, Korea; 79 Institute of Computer Science, Polish Academy of Sciences, 01-248 Warsawa, Poland; 80 Genome Biology Unit, European Bioinformatics Institute (EMBL-EBI), 69117 Heidelberg, Germany; 81 Harvard University, Cambridge, MA 02138 USA; 82 Memorial Sloan Kettering Cancer Center, New York, NY 10065 USA; 83 Department of Molecular Biophysics and Biochemistry, New Haven, CT 06520 USA; 84 Program in Computational Biology and Bioinformatics, New Haven, CT 06520 USA; 85 The Jackson Laboratory for Genomic Medicine, Farmington, CT 06032 USA; 86 Yale University, New Haven, CT 06520 USA; 87 Department of Information Technology, Ghent University, 9000 Ghent, Belgium; 88 Department of Plant Biotechnology and Bioinformatics, Ghent University, 9000 Ghent, Belgium; 89 Yale School of Medicine, Yale University, New Haven, CT 06520 USA; 90 Division of Hematology-Oncology, Samsung Medical Center, Sungkyunkwan University School of Medicine, Seoul, 06351 South Korea; 91 Samsung Advanced Institute for Health Sciences and Technology, Sungkyunkwan University School of Medicine, Seoul, 06351 South Korea; 92 Cheonan Industry-Academic Collaboration Foundation, Sangmyung University, Cheonan, 31066 South Korea; 93 Spanish National Cancer Research Centre, 28029 Madrid, Spain; 94 Bern Center for Precision Medicine, University Hospital of Bern, University of Bern, 3008 Bern, Switzerland; 95 Englander Institute for Precision Medicine, Weill Cornell Medicine and NewYork Presbyterian Hospital, New York, NY 10021 USA; 96 Pathology and Laboratory, Weill Cornell Medical College, New York, NY 10021 USA; 97 Vall d’Hebron Institute of Oncology: VHIO, 08035 Barcelona, Spain; 98 National Centre for Biological Sciences, Tata Institute of Fundamental Research, Bangalore, 560065 India; 99 Indiana University, Bloomington, IN 47405 USA; 100 Vancouver Prostate Centre, Vancouver, BC V6H 3Z6 Canada; 101 cBio Center, Dana-Farber Cancer Institute, Harvard Medical School, Boston, MA 02115 USA; 102 Department of Cell Biology, Harvard Medical School, Boston, MA 02115 USA; 103 Department of Cancer Biology, Dana-Farber Cancer Institute, Boston, MA 02215 USA; 104 Peter MacCallum Cancer Centre and University of Melbourne, Melbourne, VIC 3000 Australia; 105 cBio Center, Dana-Farber Cancer Institute, Boston, MA 02215 USA; 106 Finsen Laboratory and Biotech Research & Innovation Centre (BRIC), University of Copenhagen, 2200 Copenhagen, Denmark; 107 Department of Genetics, Stanford University School of Medicine, Stanford, CA 94305 USA; 108 CREST, Japan Science and Technology Agency, Tokyo, 113-0033 Japan; 109 Department of Medical Science Mathematics, Tokyo Medical and Dental University, Bunkyo-ku, Tokyo 113-8510 Japan; 110 Laboratory for Medical Science Mathematics, Department of Biological Sciences, Graduate School of Science, The University of Tokyo, Bunkyo-ku, Tokyo 113-0033 Japan; 111 Department of Oncology-Pathology, Science for Life Laboratory, Karolinska Institute, Stockholm, Sweden; 112 Department of Gene Technology, Tallinn University of Technology, 12616 Tallinn, Estonia; 113 Genetics & Genome Biology Program, SickKids Research Institute, The Hospital for Sick Children, Toronto, ON M5G 1X8 Canada; 114 Barcelona Supercomputing Center (BSC), 08034 Barcelona, Spain; 115 Institució Catalana de Recerca i Estudis Avançats (ICREA), 08010 Barcelona, Spain; 116 Department of Clinical and Molecular Medicine, Faculty of Medicine and Health Sciences, Norwegian University of Science and Technology, 7030 Trondheim, Norway; 117 Department of Information Technology, Ghent University, Interuniversitair Micro-Electronica Centrum (IMEC), 9000 Ghent, Belgium; 118 Science for Life Laboratory, Department of Immunology, Genetics and Pathology, Uppsala University, SE-75108 Uppsala, Sweden; 119 School of Computer Science and Technology, Xi’an Jiaotong University, 710048 Xi’an, China; 120 School of Electronic and Information Engineering, Xi’an Jiaotong University, 710048 Xi’an, China; 121 The McDonnell Genome Institute at Washington University, St Louis, MO 63108 USA; 122 Department of Urology, Charité Universitätsmedizin Berlin, 10117 Berlin, Germany; 123 Department of Molecular and Human Genetics, Baylor College of Medicine, Houston, TX 77030 USA; 124 Human Genome Sequencing Center, Baylor College of Medicine, Houston, TX 77030 USA; 125 Oregon Health & Sciences University, Portland, OR 97239 USA; 126 Department of Medicine and Therapeutics, The Chinese University of Hong Kong, Shatin, NT, Hong Kong China; 127 Second Military Medical University, Shanghai, 200433 China; 128 The University of Texas Health Science Center at Houston, Houston, TX 77030 USA; 129 Department of Biomedical Informatics, College of Medicine, The Ohio State University, Columbus, OH 43210 USA; 130 The Ohio State University Comprehensive Cancer Center (OSUCCC – James), Columbus, OH 43210 USA; 131 School of Biomedical Informatics, The University of Texas Health Science Center at Houston, Houston, TX 77030 USA; 132 Department of Biochemistry and Molecular Genetics, Feinberg School of Medicine, Northwestern University, Chicago, IL 60637 USA; 133 Institute of Molecular Life Sciences and Swiss Institute of Bioinformatics, University of Zurich, 8057 Zurich, Switzerland; 134 Present Address: The Jackson Laboratory for Genomic Medicine, Farmington, CT 06032 USA; 200 Applied Tumor Genomics Research Program, Research Programs Unit, University of Helsinki, Helsinki, Finland; 201 Wellcome Sanger Institute, Wellcome Genome Campus, Hinxton, UK; 202 Memorial Sloan Kettering Cancer Center, New York, NY USA; 203 Genome Science Division, Research Center for Advanced Science and Technology, University of Tokyo, Tokyo, Japan; 204 Department of Surgery, University of Chicago, Chicago, IL USA; 205 Department of Surgery, Division of Hepatobiliary and Pancreatic Surgery, School of Medicine, Keimyung University Dongsan Medical Center, Daegu, South Korea; 206 Department of Oncology, Gil Medical Center, Gachon University, Incheon, South Korea; 207 Hiroshima University, Hiroshima, Japan; 208 Department of Bioinformatics and Computational Biology, The University of Texas MD Anderson Cancer Center, Houston, TX USA; 209 University of Texas MD Anderson Cancer Center, Houston, TX USA; 210 King Faisal Specialist Hospital and Research Centre, Al Maather, Riyadh, Saudi Arabia; 211 Bioinformatics Unit, Spanish National Cancer Research Centre (CNIO), Madrid, Spain; 212 Bioinformatics Core Facility, University Medical Center Hamburg, Hamburg, Germany; 213 Heinrich Pette Institute, Leibniz Institute for Experimental Virology, Hamburg, Germany; 214 Ontario Tumour Bank, Ontario Institute for Cancer Research, Toronto, ON Canada; 215 Department of Pathology, The University of Texas MD Anderson Cancer Center, Houston, TX USA; 216 Laboratory of Pathology, Center for Cancer Research, National Cancer Institute, Bethesda, MD USA; 217 Department of Cellular and Molecular Medicine and Department of Bioengineering, University of California San Diego, La Jolla, CA USA; 218 UC San Diego Moores Cancer Center, San Diego, CA USA; 219 Canada’s Michael Smith Genome Sciences Centre, BC Cancer, Vancouver, BC Canada; 220 Sir Peter MacCallum Department of Oncology, Peter MacCallum Cancer Centre, University of Melbourne, Melbourne, VIC Australia; 221 Centre for Research in Molecular Medicine and Chronic Diseases (CiMUS), Universidade de Santiago de Compostela, Santiago de Compostela, Spain; 222 Department of Zoology, Genetics and Physical Anthropology, (CiMUS), Universidade de Santiago de Compostela, Santiago de Compostela, Spain; 223 The Biomedical Research Centre (CINBIO), Universidade de Vigo, Vigo, Spain; 224 Royal National Orthopaedic Hospital - Bolsover, London, UK; 225 Department of Genomic Medicine, The University of Texas MD Anderson Cancer Center, Houston, TX USA; 226 Quantitative and Computational Biosciences Graduate Program, Baylor College of Medicine, Houston, TX USA; 227 The Jackson Laboratory for Genomic Medicine, Farmington, CT USA; 228 Genome Informatics Program, Ontario Institute for Cancer Research, Toronto, ON Canada; 229 Institute of Human Genetics, Christian-Albrechts-University, Kiel, Germany; 230 Institute of Human Genetics, Ulm University and Ulm University Medical Center, Ulm, Germany; 231 Queensland Centre for Medical Genomics, Institute for Molecular Bioscience, University of Queensland, St. Lucia, Brisbane, QLD Australia; 232 Salford Royal NHS Foundation Trust, Salford, UK; 233 Department of Surgery, Pancreas Institute, University and Hospital Trust of Verona, Verona, Italy; 234 Molecular and Medical Genetics, OHSU Knight Cancer Institute, Oregon Health and Science University, Portland, OR USA; 235 Department of Molecular Oncology, BC Cancer Research Centre, Vancouver, BC Canada; 236 The McDonnell Genome Institute at Washington University, St. Louis, MO USA; 237 University College London, London, UK; 238 Division of Cancer Genomics, National Cancer Center Research Institute, National Cancer Center, Tokyo, Japan; 239 DLR Project Management Agency, Bonn, Germany; 240 Tokyo Women’s Medical University, Tokyo, Japan; 241 Center for Molecular Oncology, Memorial Sloan Kettering Cancer Center, New York, NY USA; 242 Los Alamos National Laboratory, Los Alamos, NM USA; 243 Department of Pathology, University Health Network, Toronto General Hospital, Toronto, ON Canada; 244 Nottingham University Hospitals NHS Trust, Nottingham, UK; 245 Epigenomics and Cancer Risk Factors, German Cancer Research Center (DKFZ), Heidelberg, Germany; 246 Computational Biology Program, Ontario Institute for Cancer Research, Toronto, ON Canada; 247 Department of Molecular Genetics, University of Toronto, Toronto, ON Canada; 248 Vector Institute, Toronto, ON Canada; 249 Hematopathology Section, Institute of Pathology, Christian-Albrechts-University, Kiel, Germany; 250 Department of Pathology and Laboratory Medicine, School of Medicine, University of North Carolina at Chapel Hill, Chapel Hill, NC USA; 251 Department of Cancer Genetics, Institute for Cancer Research, Oslo University Hospital, The Norwegian Radium Hospital, Oslo, Norway; 252 Pathology, Hospital Clinic, Institut d’Investigacions Biomèdiques August Pi i Sunyer (IDIBAPS), University of Barcelona, Barcelona, Spain; 253 Department of Veterinary Medicine, Transmissible Cancer Group, University of Cambridge, Cambridge, UK; 254 Alvin J. Siteman Cancer Center, Washington University School of Medicine, St. Louis, MO USA; 255 Wolfson Wohl Cancer Research Centre, Institute of Cancer Sciences, University of Glasgow, Glasgow, UK; 256 Lineberger Comprehensive Cancer Center, University of North Carolina at Chapel Hill, Chapel Hill, NC USA; 257 Broad Institute of MIT and Harvard, Cambridge, MA USA; 258 Dana-Farber/Boston Children’s Cancer and Blood Disorders Center, Boston, MA USA; 259 Department of Pediatrics, Harvard Medical School, Boston, MA USA; 260 Leeds Institute of Medical Research @ St. James’s, University of Leeds, St. James’s University Hospital, Leeds, UK; 261 Department of Pathology and Diagnostics, University and Hospital Trust of Verona, Verona, Italy; 262 Department of Surgery, Princess Alexandra Hospital, Brisbane, QLD Australia; 263 Surgical Oncology Group, Diamantina Institute, University of Queensland, Brisbane, QLD Australia; 264 Department of Population and Quantitative Health Sciences, Case Western Reserve University School of Medicine, Cleveland, OH USA; 265 Research Health Analytics and Informatics, University Hospitals Cleveland Medical Center, Cleveland, OH USA; 266 Gloucester Royal Hospital, Gloucester, UK; 267 European Molecular Biology Laboratory, European Bioinformatics Institute (EMBL-EBI), Cambridge, UK; 268 Diagnostic Development, Ontario Institute for Cancer Research, Toronto, ON Canada; 269 Barcelona Supercomputing Center (BSC), Barcelona, Spain; 270 Arnie Charbonneau Cancer Institute, University of Calgary, Calgary, AB Canada; 271 Departments of Surgery and Oncology, University of Calgary, Calgary, AB Canada; 272 Department of Pathology, Oslo University Hospital, The Norwegian Radium Hospital, Oslo, Norway; 273 PanCuRx Translational Research Initiative, Ontario Institute for Cancer Research, Toronto, ON Canada; 274 Department of Oncology, Sidney Kimmel Comprehensive Cancer Center at Johns Hopkins University School of Medicine, Baltimore, MD USA; 275 University Hospital Southampton NHS Foundation Trust, Southampton, UK; 276 Royal Stoke University Hospital, Stoke-on-Trent, UK; 277 Genome Sequence Informatics, Ontario Institute for Cancer Research, Toronto, ON Canada; 278 Human Longevity Inc, San Diego, CA USA; 279 Olivia Newton-John Cancer Research Institute, La Trobe University, Heidelberg, VIC Australia; 280 Computer Network Information Center, Chinese Academy of Sciences, Beijing, China; 281 Genome Canada, Ottawa, ON Canada; 282 CNAG-CRG, Centre for Genomic Regulation (CRG), Barcelona Institute of Science and Technology (BIST), Barcelona, Spain; 283 Universitat Pompeu Fabra (UPF), Barcelona, Spain; 284 Buck Institute for Research on Aging, Novato, CA USA; 285 Duke University Medical Center, Durham, NC USA; 286 Department of Human Genetics, Hannover Medical School, Hannover, Germany; 287 Center for Bioinformatics and Functional Genomics, Cedars-Sinai Medical Center, Los Angeles, CA USA; 288 Department of Biomedical Sciences, Cedars-Sinai Medical Center, Los Angeles, CA USA; 289 The Hebrew University Faculty of Medicine, Jerusalem, Israel; 290 Barts Cancer Institute, Barts and the London School of Medicine and Dentistry, Queen Mary University of London, London, UK; 291 Department of Computer Science, Bioinformatics Group, University of Leipzig, Leipzig, Germany; 292 Interdisciplinary Center for Bioinformatics, University of Leipzig, Leipzig, Germany; 293 Transcriptome Bioinformatics, LIFE Research Center for Civilization Diseases, University of Leipzig, Leipzig, Germany; 294 Department of Medical Oncology, Dana-Farber Cancer Institute, Boston, MA USA; 295 Department of Cancer Biology, Dana-Farber Cancer Institute, Boston, MA USA; 296 Harvard Medical School, Boston, MA USA; 297 USC Norris Comprehensive Cancer Center, University of Southern California, Los Angeles, CA USA; 298 Department of Diagnostics and Public Health, University and Hospital Trust of Verona, Verona, Italy; 299 Department of Mathematics, Aarhus University, Aarhus, Denmark; 300 Department of Molecular Medicine (MOMA), Aarhus University Hospital, Aarhus N, Denmark; 301 Instituto Carlos Slim de la Salud, Mexico City, Mexico; 302 Department of Medical Biophysics, University of Toronto, Toronto, ON Canada; 303 Cancer Division, Garvan Institute of Medical Research, Kinghorn Cancer Centre, University of New South Wales (UNSW Sydney), Sydney, NSW Australia; 304 South Western Sydney Clinical School, Faculty of Medicine, University of New South Wales (UNSW Sydney), Liverpool, NSW Australia; 305 West of Scotland Pancreatic Unit, Glasgow Royal Infirmary, Glasgow, UK; 306 Center for Digital Health, Berlin Institute of Health and Charitè - Universitätsmedizin Berlin, Berlin, Germany; 307 Heidelberg Center for Personalized Oncology (DKFZ-HIPO), German Cancer Research Center (DKFZ), Heidelberg, Germany; 308 The Preston Robert Tisch Brain Tumor Center, Duke University Medical Center, Durham, NC USA; 309 Massachusetts General Hospital, Boston, MA USA; 310 National Institute of Biomedical Genomics, Kalyani, West Bengal India; 311 Institute of Clinical Medicine and Institute of Oral Biology, University of Oslo, Oslo, Norway; 312 University of North Carolina at Chapel Hill, Chapel Hill, NC USA; 313 ARC-Net Centre for Applied Research on Cancer, University and Hospital Trust of Verona, Verona, Italy; 314 The Institute of Cancer Research, London, UK; 315 Centre for Computational Biology, Duke-NUS Medical School, Singapore, Singapore; 316 Programme in Cancer and Stem Cell Biology, Duke-NUS Medical School, Singapore, Singapore; 317 Division of Oncology and Pathology, Department of Clinical Sciences Lund, Lund University, Lund, Sweden; 318 Department of Pediatric Oncology, Hematology and Clinical Immunology, Heinrich-Heine-University, Düsseldorf, Germany; 319 Laboratory for Medical Science Mathematics, RIKEN Center for Integrative Medical Sciences, Yokohama, Japan; 320 RIKEN Center for Integrative Medical Sciences, Yokohama, Japan; 321 Department of Internal Medicine/Hematology, Friedrich-Ebert-Hospital, Neumünster, Germany; 322 Departments of Dermatology and Pathology, Yale University, New Haven, CT USA; 323 Centre for Genomic Regulation (CRG), The Barcelona Institute of Science and Technology, Barcelona, Spain; 324 Radcliffe Department of Medicine, University of Oxford, Oxford, UK; 325 Canadian Center for Computational Genomics, McGill University, Montreal, QC Canada; 326 Department of Human Genetics, McGill University, Montreal, QC Canada; 327 Department of Human Genetics, University of California Los Angeles, Los Angeles, CA USA; 328 Department of Pharmacology, University of Toronto, Toronto, ON Canada; 329 Faculty of Medicine and Health Technology, Tampere University and Tays Cancer Center, Tampere University Hospital, Tampere, Finland; 330 Haematology, Leeds Teaching Hospitals NHS Trust, Leeds, UK; 331 Translational Research and Innovation, Centre Léon Bérard, Lyon, France; 332 Fox Chase Cancer Center, Philadelphia, PA USA; 333 International Agency for Research on Cancer, World Health Organization, Lyon, France; 334 Earlham Institute, Norwich, UK; 335 Norwich Medical School, University of East Anglia, Norwich, UK; 336 Department of Molecular Biology, Faculty of Science, Radboud Institute for Molecular Life Sciences, Radboud University, Nijmegen, HB The Netherlands; 337 CRUK Manchester Institute and Centre, Manchester, UK; 338 Department of Radiation Oncology, University of Toronto, Toronto, ON Canada; 339 Division of Cancer Sciences, Manchester Cancer Research Centre, University of Manchester, Manchester, UK; 340 Radiation Medicine Program, Princess Margaret Cancer Centre, Toronto, ON Canada; 341 Department of Pathology, Brigham and Women’s Hospital, Harvard Medical School, Boston, MA USA; 342 Department of Surgery, Division of Thoracic Surgery, The Johns Hopkins University School of Medicine, Baltimore, MD USA; 343 Division of Molecular Pathology, The Netherlands Cancer Institute, Oncode Institute, Amsterdam, CX The Netherlands; 344 Department of Biomolecular Engineering, University of California Santa Cruz, Santa Cruz, CA USA; 345 UC Santa Cruz Genomics Institute, University of California Santa Cruz, Santa Cruz, CA USA; 346 Division of Applied Bioinformatics, German Cancer Research Center (DKFZ), Heidelberg, Germany; 347 German Cancer Consortium (DKTK), German Cancer Research Center (DKFZ), Heidelberg, Germany; 348 National Center for Tumor Diseases (NCT) Heidelberg, Heidelberg, Germany; 349 Center for Biological Sequence Analysis, Department of Bio and Health Informatics, Technical University of Denmark, Lyngby, Denmark; 350 Novo Nordisk Foundation Center for Protein Research, University of Copenhagen, Copenhagen, Denmark; 351 Institute for Molecular Bioscience, University of Queensland, St. Lucia, Brisbane, QLD Australia; 352 Biomedical Engineering, Oregon Health and Science University, Portland, OR USA; 353 Division of Theoretical Bioinformatics, German Cancer Research Center (DKFZ), Heidelberg, Germany; 354 Institute of Pharmacy and Molecular Biotechnology and BioQuant, Heidelberg University, Heidelberg, Germany; 355 Federal Ministry of Education and Research, Berlin, Germany; 356 Melanoma Institute Australia, University of Sydney, Sydney, NSW Australia; 357 Pediatric Hematology and Oncology, University Hospital Muenster, Muenster, Germany; 358 Department of Pathology, Johns Hopkins University School of Medicine, Baltimore, MD USA; 359 McKusick-Nathans Institute of Genetic Medicine, Sidney Kimmel Comprehensive Cancer Center at Johns Hopkins University School of Medicine, Baltimore, MD USA; 360 Foundation Medicine, Inc, Cambridge, MA USA; 361 Department of Biomedical Data Science, Stanford University School of Medicine, Stanford, CA USA; 362 Department of Genetics, Stanford University School of Medicine, Stanford, CA USA; 363 Bakar Computational Health Sciences Institute and Department of Pediatrics, University of California, San Francisco, CA USA; 364 Institute of Clinical Medicine, Faculty of Medicine, University of Oslo, Oslo, Norway; 365 National Cancer Institute, National Institutes of Health, Bethesda, MD USA; 366 Royal Marsden NHS Foundation Trust, London and Sutton, UK; 367 Genome Biology Unit, European Molecular Biology Laboratory (EMBL), Heidelberg, Germany; 368 Department of Oncology, University of Cambridge, Cambridge, UK; 369 Li Ka Shing Centre, Cancer Research UK Cambridge Institute, University of Cambridge, Cambridge, UK; 370 Institut Gustave Roussy, Villejuif, France; 371 Cambridge University Hospitals NHS Foundation Trust, Cambridge, UK; 372 Department of Haematology, University of Cambridge, Cambridge, UK; 373 Anatomia Patológica, Hospital Clinic, Institut d’Investigacions Biomèdiques August Pi i Sunyer (IDIBAPS), University of Barcelona, Barcelona, Spain; 374 Spanish Ministry of Science and Innovation, Madrid, Spain; 375 University of Michigan Comprehensive Cancer Center, Ann Arbor, MI USA; 376 Department for BioMedical Research, University of Bern, Bern, Switzerland; 377 Department of Medical Oncology, Inselspital, University Hospital and University of Bern, Bern, Switzerland; 378 Graduate School for Cellular and Biomedical Sciences, University of Bern, Bern, Switzerland; 379 University of Pavia, Pavia, Italy; 380 University of Alabama at Birmingham, Birmingham, AL USA; 381 UHN Program in BioSpecimen Sciences, Toronto General Hospital, Toronto, ON Canada; 382 Department of Urology, Icahn School of Medicine at Mount Sinai, New York, NY USA; 383 Centre for Law and Genetics, University of Tasmania, Sandy Bay Campus, Hobart, TAS Australia; 384 Faculty of Biosciences, Heidelberg University, Heidelberg, Germany; 385 Department of Biochemistry, Microbiology and Immunology, Faculty of Medicine, University of Ottawa, Ottawa, ON Canada; 386 Division of Anatomic Pathology, Mayo Clinic, Rochester, MN USA; 387 Division of Cancer Epidemiology and Genetics, National Cancer Institute, National Institutes of Health, Bethesda, MD USA; 388 Illawarra Shoalhaven Local Health District L3 Illawarra Cancer Care Centre, Wollongong Hospital, Wollongong, NSW Australia; 389 BioForA, French National Institute for Agriculture, Food, and Environment (INRAE), ONF, Orléans, France; 390 Department of Biostatistics, Bloomberg School of Public Health, Johns Hopkins University, Baltimore, MD USA; 391 University of California San Diego, San Diego, CA USA; 392 Division of Experimental Pathology, Mayo Clinic, Rochester, MN USA; 393 Centre for Cancer Research, The Westmead Institute for Medical Research, University of Sydney, Sydney, NSW Australia; 394 Department of Gynaecological Oncology, Westmead Hospital, Sydney, NSW Australia; 395 PDXen Biosystems Inc, Seoul, South Korea; 396 Korea Advanced Institute of Science and Technology, Daejeon, South Korea; 397 Electronics and Telecommunications Research Institute, Daejeon, South Korea; 398 Institut National du Cancer (INCA), Boulogne-Billancourt, France; 399 Department of Genetics, Informatics Institute, University of Alabama at Birmingham, Birmingham, AL USA; 400 Division of Medical Oncology, National Cancer Centre, Singapore, Singapore; 401 Medical Oncology, University and Hospital Trust of Verona, Verona, Italy; 402 Department of Pediatrics, University Hospital Schleswig-Holstein, Kiel, Germany; 403 Hepatobiliary/Pancreatic Surgical Oncology Program, University Health Network, Toronto, ON Canada; 404 School of Biological Sciences, University of Auckland, Auckland, New Zealand; 405 Department of Surgery, University of Melbourne, Parkville, VIC Australia; 406 The Murdoch Children’s Research Institute, Royal Children’s Hospital, Parkville, VIC Australia; 407 Walter and Eliza Hall Institute, Parkville, VIC Australia; 408 Vancouver Prostate Centre, Vancouver, Canada; 409 Lunenfeld-Tanenbaum Research Institute, Mount Sinai Hospital, Toronto, ON Canada; 410 University of East Anglia, Norwich, UK; 411 Norfolk and Norwich University Hospital NHS Trust, Norwich, UK; 412 Victorian Institute of Forensic Medicine, Southbank, VIC Australia; 413 Department of Biomedical Informatics, Harvard Medical School, Boston, MA USA; 414 Department of Chemistry, Centre for Molecular Science Informatics, University of Cambridge, Cambridge, UK; 415 Ludwig Center at Harvard Medical School, Boston, MA USA; 416 Human Genome Sequencing Center, Baylor College of Medicine, Houston, TX USA; 417 Peter MacCallum Cancer Centre, University of Melbourne, Melbourne, VIC Australia; 418 Physics Division, Optimization and Systems Biology Lab, Massachusetts General Hospital, Boston, MA USA; 419 Department of Medicine, Baylor College of Medicine, Houston, TX USA; 420 University of Cologne, Cologne, Germany; 421 International Genomics Consortium, Phoenix, AZ USA; 422 Genomics Research Program, Ontario Institute for Cancer Research, Toronto, ON Canada; 423 Barking Havering and Redbridge University Hospitals NHS Trust, Romford, UK; 424 Children’s Hospital at Westmead, University of Sydney, Sydney, NSW Australia; 425 Department of Medicine, Section of Endocrinology, University and Hospital Trust of Verona, Verona, Italy; 426 Computational Biology Center, Memorial Sloan Kettering Cancer Center, New York, NY USA; 427 Department of Biology, ETH Zurich, Zürich, Switzerland; 428 Department of Computer Science, ETH Zurich, Zurich, Switzerland; 429 SIB Swiss Institute of Bioinformatics, Lausanne, Switzerland; 430 Weill Cornell Medical College, New York, NY USA; 431 Academic Department of Medical Genetics, University of Cambridge, Addenbrooke’s Hospital, Cambridge, UK; 432 MRC Cancer Unit, University of Cambridge, Cambridge, UK; 433 Departments of Pediatrics and Genetics, University of North Carolina at Chapel Hill, Chapel Hill, NC USA; 434 Seven Bridges Genomics, Charlestown, MA USA; 435 Annai Systems, Inc, Carlsbad, CA USA; 436 Department of Pathology, General Hospital of Treviso, Department of Medicine, University of Padua, Treviso, Italy; 437 Department of Computational Biology, University of Lausanne, Lausanne, Switzerland; 438 Department of Genetic Medicine and Development, University of Geneva Medical School, Geneva, CH Switzerland; 439 Swiss Institute of Bioinformatics, University of Geneva, Geneva, CH Switzerland; 440 The Francis Crick Institute, London, UK; 441 University of Leuven, Leuven, Belgium; 442 Institute of Medical Genetics and Applied Genomics, University of Tübingen, Tübingen, Germany; 443 Computational and Systems Biology, Genome Institute of Singapore, Singapore, Singapore; 444 School of Computing, National University of Singapore, Singapore, Singapore; 445 Big Data Institute, Li Ka Shing Centre, University of Oxford, Oxford, UK; 446 Biomedical Data Science Laboratory, Francis Crick Institute, London, UK; 447 Bioinformatics Group, Department of Computer Science, University College London, London, UK; 448 The Edward S. Rogers Sr. Department of Electrical and Computer Engineering, University of Toronto, Toronto, ON Canada; 449 Breast Cancer Translational Research Laboratory JC Heuson, Institut Jules Bordet, Brussels, Belgium; 450 Department of Oncology, Laboratory for Translational Breast Cancer Research, KU Leuven, Leuven, Belgium; 451 Institute for Research in Biomedicine (IRB Barcelona), The Barcelona Institute of Science and Technology, Barcelona, Spain; 452 Research Program on Biomedical Informatics, Universitat Pompeu Fabra, Barcelona, Spain; 453 Division of Medical Oncology, Princess Margaret Cancer Centre, Toronto, ON Canada; 454 Department of Physiology and Biophysics, Weill Cornell Medicine, New York, NY USA; 455 Institute for Computational Biomedicine, Weill Cornell Medicine, New York, NY USA; 456 Department of Pathology, UPMC Shadyside, Pittsburgh, PA USA; 457 Independent Consultant, Wellesley, USA; 458 Department of Cell and Molecular Biology, Science for Life Laboratory, Uppsala University, Uppsala, Sweden; 459 Department of Medicine and Department of Genetics, Washington University School of Medicine, St. Louis, St. Louis, MO USA; 460 Hefei University of Technology, Anhui, China; 461 Translational Cancer Research Unit, GZA Hospitals St.-Augustinus, Center for Oncological Research, Faculty of Medicine and Health Sciences, University of Antwerp, Antwerp, Belgium; 462 Simon Fraser University, Burnaby, BC Canada; 463 University of Pennsylvania, Philadelphia, PA USA; 464 Faculty of Science and Technology, University of Vic—Central University of Catalonia (UVic-UCC), Vic, Spain; 465 The Wellcome Trust, London, UK; 466 The Hospital for Sick Children, Toronto, ON Canada; 467 Department of Pathology, Queen Elizabeth University Hospital, Glasgow, UK; 468 Department of Genetics and Computational Biology, QIMR Berghofer Medical Research Institute, Brisbane, QLD Australia; 469 Department of Oncology, Centre for Cancer Genetic Epidemiology, University of Cambridge, Cambridge, UK; 470 Department of Public Health and Primary Care, Centre for Cancer Genetic Epidemiology, University of Cambridge, Cambridge, UK; 471 Prostate Cancer Canada, Toronto, ON Canada; 472 University of Cambridge, Cambridge, UK; 473 Department of Laboratory Medicine, Translational Cancer Research, Lund University Cancer Center at Medicon Village, Lund University, Lund, Sweden; 474 Heidelberg University, Heidelberg, Germany; 475 New BIH Digital Health Center, Berlin Institute of Health (BIH) and Charité - Universitätsmedizin Berlin, Berlin, Germany; 476 CIBER Epidemiología y Salud Pública (CIBERESP), Madrid, Spain; 477 Research Group on Statistics, Econometrics and Health (GRECS), UdG, Barcelona, Spain; 478 Quantitative Genomics Laboratories (qGenomics), Barcelona, Spain; 479 Icelandic Cancer Registry, Icelandic Cancer Society, Reykjavik, Iceland; 480 State Key Laboratory of Cancer Biology, and Xijing Hospital of Digestive Diseases, Fourth Military Medical University, Shaanxi, China; 481 Department of Medicine (DIMED), Surgical Pathology Unit, University of Padua, Padua, Italy; 482 Rigshospitalet, Copenhagen, Denmark; 483 Center for Cancer Genomics, National Cancer Institute, National Institutes of Health, Bethesda, MD USA; 484 Department of Biochemistry and Molecular Medicine, University of Montreal, Montreal, QC Canada; 485 Australian Institute of Tropical Health and Medicine, James Cook University, Douglas, QLD Australia; 486 Department of Neuro-Oncology, Istituto Neurologico Besta, Milano, Italy; 487 Bioplatforms Australia, North Ryde, NSW Australia; 488 Department of Pathology (Research), University College London Cancer Institute, London, UK; 489 Department of Surgical Oncology, Princess Margaret Cancer Centre, Toronto, ON Canada; 490 Department of Medical Oncology, Josephine Nefkens Institute and Cancer Genomics Centre, Erasmus Medical Center, Rotterdam, CN The Netherlands; 491 The University of Queensland Thoracic Research Centre, The Prince Charles Hospital, Brisbane, QLD Australia; 492 CIBIO/InBIO - Research Center in Biodiversity and Genetic Resources, Universidade do Porto, Vairão, Portugal; 493 HCA Laboratories, London, UK; 494 University of Liverpool, Liverpool, UK; 495 The Azrieli Faculty of Medicine, Bar-Ilan University, Safed, Israel; 496 Department of Neurosurgery, University of Florida, Gainesville, FL USA; 497 Department of Pathology, Graduate School of Medicine, University of Tokyo, Tokyo, Japan; 498 University of Milano Bicocca, Monza, Italy; 499 BGI-Shenzhen, Shenzhen, China; 500 Department of Pathology, Oslo University Hospital Ulleval, Oslo, Norway; 501 Center for Biomedical Informatics, Harvard Medical School, Boston, MA USA; 502 Department Biochemistry and Molecular Biomedicine, University of Barcelona, Barcelona, Spain; 503 Office of Cancer Genomics, National Cancer Institute, National Institutes of Health, Bethesda, MD USA; 504 Cancer Epigenomics, German Cancer Research Center (DKFZ), Heidelberg, Germany; 505 Department of Cancer Biology, The University of Texas MD Anderson Cancer Center, Houston, TX USA; 506 Department of Surgical Oncology, The University of Texas MD Anderson Cancer Center, Houston, TX USA; 507 Department of Computer Science, Yale University, New Haven, CT USA; 508 Department of Molecular Biophysics and Biochemistry, Yale University, New Haven, CT USA; 509 Program in Computational Biology and Bioinformatics, Yale University, New Haven, CT USA; 510 Center for Cancer Research, Massachusetts General Hospital, Boston, MA USA; 511 Department of Pathology, Massachusetts General Hospital, Boston, MA USA; 512 Department of Pathology, Memorial Sloan Kettering Cancer Center, New York, NY USA; 513 Division of Gastroenterology and Hepatology, Mayo Clinic, Rochester, MN USA; 514 University of Sydney, Sydney, NSW Australia; 515 University of Oxford, Oxford, UK; 516 Department of Surgery, Academic Urology Group, University of Cambridge, Cambridge, UK; 517 Department of Medicine II, University of Würzburg, Wuerzburg, Germany; 518 Sylvester Comprehensive Cancer Center, University of Miami, Miami, FL USA; 519 Institut Hospital del Mar d’Investigacions Mèdiques (IMIM), Barcelona, Spain; 520 Genome Integrity and Structural Biology Laboratory, National Institute of Environmental Health Sciences (NIEHS), Durham, NC USA; 521 St. Thomas’s Hospital, London, UK; 522 Osaka International Cancer Center, Osaka, Japan; 523 Department of Pathology, Skåne University Hospital, Lund University, Lund, Sweden; 524 Department of Medical Oncology, Beatson West of Scotland Cancer Centre, Glasgow, UK; 525 National Human Genome Research Institute, National Institutes of Health, Bethesda, MD USA; 526 Centre for Cancer Research, Victorian Comprehensive Cancer Centre, University of Melbourne, Melbourne, VIC Australia; 527 Department of Medicine, Section of Hematology/Oncology, University of Chicago, Chicago, IL USA; 528 German Center for Infection Research (DZIF), Partner Site Hamburg-Borstel-Lübeck-Riems, Hamburg, Germany; 529 Bioinformatics Research Centre (BiRC), Aarhus University, Aarhus, Denmark; 530 Department of Biotechnology, Ministry of Science and Technology, Government of India, New Delhi, Delhi India; 531 National Cancer Centre Singapore, Singapore, Singapore; 532 Brandeis University, Waltham, MA USA; 533 Department of Urologic Sciences, University of British Columbia, Vancouver, BC Canada; 534 Department of Internal Medicine, Stanford University, Stanford, CA USA; 535 The University of Texas Health Science Center at Houston, Houston, TX USA; 536 Imperial College NHS Trust, Imperial College, London, INY UK; 537 Senckenberg Institute of Pathology, University of Frankfurt Medical School, Frankfurt, Germany; 538 Department of Medicine, Division of Biomedical Informatics, UC San Diego School of Medicine, San Diego, CA USA; 539 Center for Precision Health, School of Biomedical Informatics, The University of Texas Health Science Center, Houston, TX USA; 540 Oxford Nanopore Technologies, New York, NY USA; 541 Institute of Medical Science, University of Tokyo, Tokyo, Japan; 542 Howard Hughes Medical Institute, University of California Santa Cruz, Santa Cruz, CA USA; 543 Wakayama Medical University, Wakayama, Japan; 544 Department of Internal Medicine, Division of Medical Oncology, Lineberger Comprehensive Cancer Center, University of North Carolina at Chapel Hill, Chapel Hill, NC USA; 545 University of Tennessee Health Science Center for Cancer Research, Memphis, TN USA; 546 Department of Histopathology, Salford Royal NHS Foundation Trust, Salford, UK; 547 Faculty of Biology, Medicine and Health, University of Manchester, Manchester, UK; 548 BIOPIC, ICG and College of Life Sciences, Peking University, Beijing, China; 549 Peking-Tsinghua Center for Life Sciences, Peking University, Beijing, China; 550 Children’s Hospital of Philadelphia, Philadelphia, PA USA; 551 Department of Bioinformatics and Computational Biology and Department of Systems Biology, The University of Texas MD Anderson Cancer Center, Houston, TX USA; 552 Karolinska Institute, Stockholm, Sweden; 553 The Donnelly Centre, University of Toronto, Toronto, ON Canada; 554 Department of Medical Genetics, College of Medicine, Hallym University, Chuncheon, South Korea; 555 Department of Experimental and Health Sciences, Institute of Evolutionary Biology (UPF-CSIC), Universitat Pompeu Fabra, Barcelona, Spain; 556 Health Data Science Unit, University Clinics, Heidelberg, Germany; 557 Massachusetts General Hospital Center for Cancer Research, Charlestown, MA USA; 558 Hokkaido University, Sapporo, Japan; 559 Department of Pathology and Clinical Laboratory, National Cancer Center Hospital, Tokyo, Japan; 560 Department of Genetics, University of North Carolina at Chapel Hill, Chapel Hill, NC USA; 561 Computational Biology, Leibniz Institute on Aging - Fritz Lipmann Institute (FLI), Jena, Germany; 562 University of Melbourne Centre for Cancer Research, Melbourne, VIC Australia; 563 University of Nebraska Medical Center, Omaha, NE USA; 564 Syntekabio Inc, Daejeon, South Korea; 565 Department of Pathology, Academic Medical Center, Amsterdam, AZ The Netherlands; 566 China National GeneBank-Shenzhen, Shenzhen, China; 567 Division of Molecular Genetics, German Cancer Research Center (DKFZ), Heidelberg, Germany; 568 Division of Life Science and Applied Genomics Center, Hong Kong University of Science and Technology, Clear Water Bay, Hong Kong, China; 569 Icahn School of Medicine at Mount Sinai, New York, NY USA; 570 Geneplus-Shenzhen, Shenzhen, China; 571 School of Computer Science and Technology, Xi’an Jiaotong University, Xi’an, China; 572 AbbVie, North Chicago, IL USA; 573 Institute of Pathology, Charité – University Medicine Berlin, Berlin, Germany; 574 Centre for Translational and Applied Genomics, British Columbia Cancer Agency, Vancouver, BC Canada; 575 Edinburgh Royal Infirmary, Edinburgh, UK; 576 Berlin Institute for Medical Systems Biology, Max Delbrück Center for Molecular Medicine, Berlin, Germany; 577 Department of Pediatric Immunology, Hematology and Oncology, University Hospital, Heidelberg, Germany; 578 German Cancer Research Center (DKFZ), Heidelberg, Germany; 579 Heidelberg Institute for Stem Cell Technology and Experimental Medicine (HI-STEM), Heidelberg, Germany; 580 Institute for Computational Biomedicine, Weill Cornell Medical College, New York, NY USA; 581 New York Genome Center, New York, NY USA; 582 Department of Urology, James Buchanan Brady Urological Institute, Johns Hopkins University School of Medicine, Baltimore, MD USA; 583 Department of Preventive Medicine, Graduate School of Medicine, The University of Tokyo, Tokyo, Japan; 584 Department of Molecular and Cellular Biology, Baylor College of Medicine, Houston, TX USA; 585 Department of Pathology and Immunology, Baylor College of Medicine, Houston, TX USA; 586 Michael E. DeBakey Veterans Affairs Medical Center, Houston, TX USA; 587 Technical University of Denmark, Lyngby, Denmark; 588 Department of Pathology, College of Medicine, Hanyang University, Seoul, South Korea; 589 Academic Unit of Surgery, School of Medicine, College of Medical, Veterinary and Life Sciences, University of Glasgow, Glasgow Royal Infirmary, Glasgow, UK; 590 Department of Pathology, Asan Medical Center, College of Medicine, Ulsan University, Songpa-gu, Seoul South Korea; 591 Science Writer, Garrett Park, MD USA; 592 International Cancer Genome Consortium (ICGC)/ICGC Accelerating Research in Genomic Oncology (ARGO) Secretariat, Ontario Institute for Cancer Research, Toronto, ON Canada; 593 University of Ljubljana, Ljubljana, Slovenia; 594 Department of Public Health Sciences, University of Chicago, Chicago, IL USA; 595 Research Institute, NorthShore University HealthSystem, Evanston, IL USA; 596 Department for Biomedical Research, University of Bern, Bern, Switzerland; 597 Centre of Genomics and Policy, McGill University and Génome Québec Innovation Centre, Montreal, QC Canada; 598 Carolina Center for Genome Sciences, University of North Carolina at Chapel Hill, Chapel Hill, NC USA; 599 Hopp Children’s Cancer Center (KiTZ), Heidelberg, Germany; 600 Pediatric Glioma Research Group, German Cancer Research Center (DKFZ), Heidelberg, Germany; 601 Cancer Research UK, London, UK; 602 Indivumed GmbH, Hamburg, Germany; 603 Genome Integration Data Center, Syntekabio, Inc, Daejeon, South Korea; 604 University Hospital Zurich, Zurich, Switzerland; 605 Clinical Bioinformatics, Swiss Institute of Bioinformatics, Geneva, Switzerland; 606 Institute for Pathology and Molecular Pathology, University Hospital Zurich, Zurich, Switzerland; 607 Institute of Molecular Life Sciences, University of Zurich, Zurich, Switzerland; 608 MRC Human Genetics Unit, MRC IGMM, University of Edinburgh, Edinburgh, UK; 609 Women’s Cancer Program at the Samuel Oschin Comprehensive Cancer Institute, Cedars-Sinai Medical Center, Los Angeles, CA USA; 610 Department of Biology, Bioinformatics Group, Division of Molecular Biology, Faculty of Science, University of Zagreb, Zagreb, Croatia; 611 Department for Internal Medicine II, University Hospital Schleswig-Holstein, Kiel, Germany; 612 Genetics and Molecular Pathology, SA Pathology, Adelaide, SA Australia; 613 Department of Gastric Surgery, National Cancer Center Hospital, Tokyo, Japan; 614 Department of Bioinformatics, Division of Cancer Genomics, National Cancer Center Research Institute, Tokyo, Japan; 615 A.A. Kharkevich Institute of Information Transmission Problems, Moscow, Russia; 616 Oncology and Immunology, Dmitry Rogachev National Research Center of Pediatric Hematology, Moscow, Russia; 617 Skolkovo Institute of Science and Technology, Moscow, Russia; 618 Department of Surgery, The George Washington University, School of Medicine and Health Science, Washington, DC USA; 619 Endocrine Oncology Branch, Center for Cancer Research, National Cancer Institute, National Institutes of Health, Bethesda, MD USA; 620 Melanoma Institute Australia, Macquarie University, Sydney, NSW Australia; 621 MIT Computer Science and Artificial Intelligence Laboratory, Massachusetts Institute of Technology, Cambridge, MA USA; 622 Tissue Pathology and Diagnostic Oncology, Royal Prince Alfred Hospital, Sydney, NSW Australia; 623 Cholangiocarcinoma Screening and Care Program and Liver Fluke and Cholangiocarcinoma Research Centre, Faculty of Medicine, Khon Kaen University, Khon Kaen, Thailand; 624 Controlled Department and Institution, New York, NY USA; 625 Englander Institute for Precision Medicine, Weill Cornell Medicine, New York, NY USA; 626 National Cancer Center, Gyeonggi, South Korea; 627 Department of Biochemistry, College of Medicine, Ewha Womans University, Seoul, South Korea; 628 Health Sciences Department of Biomedical Informatics, University of California San Diego, La Jolla, CA USA; 629 Research Core Center, National Cancer Centre Korea, Goyang-si, South Korea; 630 Department of Health Sciences and Technology, Sungkyunkwan University School of Medicine, Seoul, South Korea; 631 Samsung Genome Institute, Seoul, South Korea; 632 Breast Oncology Program, Dana-Farber/Brigham and Women’s Cancer Center, Boston, MA USA; 633 Department of Surgery, Memorial Sloan Kettering Cancer Center, New York, NY USA; 634 Division of Breast Surgery, Brigham and Women’s Hospital, Boston, MA USA; 635 Integrative Bioinformatics Support Group, National Institute of Environmental Health Sciences (NIEHS), Durham, NC USA; 636 Department of Clinical Science, University of Bergen, Bergen, Norway; 637 Center For Medical Innovation, Seoul National University Hospital, Seoul, South Korea; 638 Department of Internal Medicine, Seoul National University Hospital, Seoul, South Korea; 639 Institute of Computer Science, Polish Academy of Sciences, Warsawa, Poland; 640 Functional and Structural Genomics, German Cancer Research Center (DKFZ), Heidelberg, Germany; 641 Laboratory of Translational Genomics, Division of Cancer Epidemiology and Genetics, National Cancer Institute, , National Institutes of Health, Bethesda, MD USA; 642 Institute for Medical Informatics Statistics and Epidemiology, University of Leipzig, Leipzig, Germany; 643 Morgan Welch Inflammatory Breast Cancer Research Program and Clinic, The University of Texas MD Anderson Cancer Center, Houston, TX USA; 644 Department of Hematology and Oncology, Georg-Augusts-University of Göttingen, Göttingen, Germany; 645 Institute of Cell Biology (Cancer Research), University of Duisburg-Essen, Essen, Germany; 646 King’s College London and Guy’s and St. Thomas’ NHS Foundation Trust, London, UK; 647 Center for Epigenetics, Van Andel Research Institute, Grand Rapids, MI USA; 648 The University of Queensland Centre for Clinical Research, Royal Brisbane and Women’s Hospital, Herston, QLD Australia; 649 Department of Pediatric Oncology and Hematology, University of Cologne, Cologne, Germany; 650 University of Düsseldorf, Düsseldorf, Germany; 651 Department of Pathology, Institut Jules Bordet, Brussels, Belgium; 652 Institute of Biomedicine, Sahlgrenska Academy at University of Gothenburg, Gothenburg, Sweden; 653 Children’s Medical Research Institute, Sydney, NSW Australia; 654 ILSbio, LLC Biobank, Chestertown, MD USA; 655 Division of Genetics and Genomics, Boston Children’s Hospital, Harvard Medical School, Boston, MA USA; 656 Institute for Bioengineering and Biopharmaceutical Research (IBBR), Hanyang University, Seoul, South Korea; 657 Department of Statistics, University of California Santa Cruz, Santa Cruz, CA USA; 658 National Genotyping Center, Institute of Biomedical Sciences, Academia Sinica, Taipei, Taiwan; 659 Department of Vertebrate Genomics/Otto Warburg Laboratory Gene Regulation and Systems Biology of Cancer, Max Planck Institute for Molecular Genetics, Berlin, Germany; 660 McGill University and Genome Quebec Innovation Centre, Montreal, QC Canada; 661 biobyte solutions GmbH, Heidelberg, Germany; 662 Gynecologic Oncology, NYU Laura and Isaac Perlmutter Cancer Center, New York University, New York, NY USA; 663 Division of Oncology, Stem Cell Biology Section, Washington University School of Medicine, St. Louis, MO USA; 664 Department of Systems Biology, The University of Texas MD Anderson Cancer Center, Houston, TX USA; 665 Harvard University, Cambridge, MA USA; 666 Urologic Oncology Branch, Center for Cancer Research, National Cancer Institute, National Institutes of Health, Bethesda, MD USA; 667 University of Oslo, Oslo, Norway; 668 University of Toronto, Toronto, ON Canada; 669 Peking University, Beijing, China; 670 School of Life Sciences, Peking University, Beijing, China; 671 Leidos Biomedical Research, Inc, McLean, VA USA; 672 Hematology, Hospital Clinic, Institut d’Investigacions Biomèdiques August Pi i Sunyer (IDIBAPS), University of Barcelona, Barcelona, Spain; 673 Second Military Medical University, Shanghai, China; 674 Chinese Cancer Genome Consortium, Shenzhen, China; 675 Department of Medical Oncology, Beijing Hospital, Beijing, China; 676 Laboratory of Molecular Oncology, Key Laboratory of Carcinogenesis and Translational Research (Ministry of Education), Peking University Cancer Hospital and Institute, Beijing, China; 677 School of Medicine/School of Mathematics and Statistics, University of St. Andrews, St, Andrews, Fife UK; 678 Institute for Systems Biology, Seattle, WA USA; 679 Department of Biochemistry and Molecular Biology, Faculty of Medicine, University Institute of Oncology-IUOPA, Oviedo, Spain; 680 Institut Bergonié, Bordeaux, France; 681 Cancer Unit, MRC University of Cambridge, Cambridge, UK; 682 Department of Pathology and Laboratory Medicine, Center for Personalized Medicine, Children’s Hospital Los Angeles, Los Angeles, CA USA; 683 John Curtin School of Medical Research, Canberra, ACT Australia; 684 MVZ Department of Oncology, PraxisClinic am Johannisplatz, Leipzig, Germany; 685 Department of Information Technology, Ghent University, Ghent, Belgium; 686 Department of Plant Biotechnology and Bioinformatics, Ghent University, Ghent, Belgium; 687 Institute for Genomic Medicine, Nationwide Children’s Hospital, Columbus, OH USA; 688 Computational Biology Program, School of Medicine, Oregon Health and Science University, Portland, OR USA; 689 Department of Surgery, Duke University, Durham, NC USA; 690 Institució Catalana de Recerca i Estudis Avançats (ICREA), Barcelona, Spain; 691 Institut Català de Paleontologia Miquel Crusafont, Universitat Autònoma de Barcelona, Barcelona, Spain; 692 University of Glasgow, Glasgow, UK; 693 Institut d’Investigacions Biomèdiques August Pi i Sunyer (IDIBAPS), Barcelona, Spain; 694 Division of Oncology, Washington University School of Medicine, St. Louis, MO USA; 695 Department of Surgery and Cancer, Imperial College, London, INY UK; 696 Applications Department, Oxford Nanopore Technologies, Oxford, UK; 697 Department of Obstetrics, Gynecology and Reproductive Services, University of California San Francisco, San Francisco, CA USA; 698 Department of Biochemistry and Molecular Medicine, University California at Davis, Sacramento, CA USA; 699 STTARR Innovation Facility, Princess Margaret Cancer Centre, Toronto, ON Canada; 700 Discipline of Surgery, Western Sydney University, Penrith, NSW Australia; 701 Yale School of Medicine, Yale University, New Haven, CT USA; 702 Department of Genetics, Lineberger Comprehensive Cancer Center, University of North Carolina at Chapel Hill, Chapel Hill, NC USA; 703 Departments of Neurology and Neurosurgery, Henry Ford Hospital, Detroit, MI USA; 704 Precision Oncology, OHSU Knight Cancer Institute, Oregon Health and Science University, Portland, OR USA; 705 Institute of Pathology, University Medical Center Hamburg-Eppendorf, Hamburg, Germany; 706 Department of Health Sciences, Faculty of Medical Sciences, Kyushu University, Fukuoka, Japan; 707 Heidelberg Academy of Sciences and Humanities, Heidelberg, Germany; 708 Department of Clinical Pathology, University of Melbourne, Melbourne, VIC, Australia; 709 Department of Pathology, Roswell Park Cancer Institute, Buffalo, NY USA; 710 Department of Computer Science, University of Helsinki, Helsinki, Finland; 711 Institute of Biotechnology, University of Helsinki, Helsinki, Finland; 712 Organismal and Evolutionary Biology Research Programme, University of Helsinki, Helsinki, Finland; 713 Department of Obstetrics and Gynecology, Division of Gynecologic Oncology, Washington University School of Medicine, St. Louis, MO USA; 714 Penrose St. Francis Health Services, Colorado Springs, CO USA; 715 Institute of Pathology, Ulm University and University Hospital of Ulm, Ulm, Germany; 716 National Cancer Center, Tokyo, Japan; 717 Genome Institute of Singapore, Singapore, Singapore; 718 32Program in Computational Biology and Bioinformatics, Yale University, New Haven, CT USA; 719 German Cancer Aid, Bonn, Germany; 720 Programme in Cancer and Stem Cell Biology, Centre for Computational Biology, Duke-NUS Medical School, Singapore, Singapore; 721 The Chinese University of Hong Kong, Shatin, NT, Hong Kong China; 722 Fourth Military Medical University, Shaanxi, China; 723 The University of Cambridge School of Clinical Medicine, Cambridge, UK; 724 St. Jude Children’s Research Hospital, Memphis, TN USA; 725 University Health Network, Princess Margaret Cancer Centre, Toronto, ON Canada; 726 Center for Biomolecular Science and Engineering, University of California Santa Cruz, Santa Cruz, CA USA; 727 Department of Medicine, University of Chicago, Chicago, IL USA; 728 Department of Neurology, Mayo Clinic, Rochester, MN USA; 729 Cambridge Oesophagogastric Centre, Cambridge University Hospitals NHS Foundation Trust, Cambridge, UK; 730 Department of Computer Science, Carleton College, Northfield, MN USA; 731 Institute of Cancer Sciences, College of Medical Veterinary and Life Sciences, University of Glasgow, Glasgow, UK; 732 Department of Epidemiology, University of Alabama at Birmingham, Birmingham, AL USA; 733 HudsonAlpha Institute for Biotechnology, Huntsville, AL USA; 734 O’Neal Comprehensive Cancer Center, University of Alabama at Birmingham, Birmingham, AL USA; 735 Department of Pathology, Keio University School of Medicine, Tokyo, Japan; 736 Department of Hepatobiliary and Pancreatic Oncology, National Cancer Center Hospital, Tokyo, Japan; 737 Sage Bionetworks, Seattle, WA USA; 738 Lymphoma Genomic Translational Research Laboratory, National Cancer Centre, Singapore, Singapore; 739 Department of Clinical Pathology, Robert-Bosch-Hospital, Stuttgart, Germany; 740 Department of Cell and Systems Biology, University of Toronto, Toronto, ON Canada; 741 Department of Biosciences and Nutrition, Karolinska Institutet, Stockholm, Sweden; 742 Center for Liver Cancer, Research Institute and Hospital, National Cancer Center, Gyeonggi, South Korea; 743 Division of Hematology-Oncology, Samsung Medical Center, Sungkyunkwan University School of Medicine, Seoul, South Korea; 744 Samsung Advanced Institute for Health Sciences and Technology, Sungkyunkwan University School of Medicine, Seoul, South Korea; 745 Cheonan Industry-Academic Collaboration Foundation, Sangmyung University, Cheonan, South Korea; 746 NYU Langone Medical Center, New York, NY USA; 747 Department of Hematology and Medical Oncology, Cleveland Clinic, Cleveland, OH USA; 748 Department of Radiation Oncology, University of California San Francisco, San Francisco, CA USA; 749 Department of Health Sciences Research, Mayo Clinic, Rochester, MN USA; 750 Helen F. Graham Cancer Center at Christiana Care Health Systems, Newark, DE USA; 751 Heidelberg University Hospital, Heidelberg, Germany; 752 CSRA Incorporated, Fairfax, VA USA; 753 Research Department of Pathology, University College London Cancer Institute, London, UK; 754 Department of Research Oncology, Guy’s Hospital, King’s Health Partners AHSC, King’s College London School of Medicine, London, UK; 755 Faculty of Medicine and Health Sciences, Macquarie University, Sydney, NSW Australia; 756 University Hospital of Minjoz, INSERM UMR 1098, Besançon, France; 757 Spanish National Cancer Research Centre, Madrid, Spain; 758 Center of Digestive Diseases and Liver Transplantation, Fundeni Clinical Institute, Bucharest, Romania; 759 Cureline, Inc, South San Francisco, CA USA; 760 St. Luke’s Cancer Centre, Royal Surrey County Hospital NHS Foundation Trust, Guildford, UK; 761 Cambridge Breast Unit, Addenbrooke’s Hospital, Cambridge University Hospital NHS Foundation Trust and NIHR Cambridge Biomedical Research Centre, Cambridge, UK; 762 East of Scotland Breast Service, Ninewells Hospital, Aberdeen, UK; 763 Department of Genetics, Microbiology and Statistics, University of Barcelona, IRSJD, IBUB, Barcelona, Spain; 764 Department of Obstetrics and Gynecology, Medical College of Wisconsin, Milwaukee, WI USA; 765 Hematology and Medical Oncology, Winship Cancer Institute of Emory University, Atlanta, GA USA; 766 Department of Computer Science, Princeton University, Princeton, NJ USA; 767 Vanderbilt Ingram Cancer Center, Vanderbilt University, Nashville, TN USA; 768 Ohio State University College of Medicine and Arthur G. James Comprehensive Cancer Center, Columbus, OH USA; 769 Department of Surgery, Yokohama City University Graduate School of Medicine, Kanagawa, Japan; 770 Division of Chromatin Networks, German Cancer Research Center (DKFZ) and BioQuant, Heidelberg, Germany; 771 Research Computing Center, University of North Carolina at Chapel Hill, Chapel Hill, NC USA; 772 School of Molecular Biosciences and Center for Reproductive Biology, Washington State University, Pullman, WA USA; 773 Finsen Laboratory and Biotech Research and Innovation Centre (BRIC), University of Copenhagen, Copenhagen, Denmark; 774 Department of Laboratory Medicine and Pathobiology, University of Toronto, Toronto, ON Canada; 775 Department of Pathology, Human Oncology and Pathogenesis Program, Memorial Sloan Kettering Cancer Center, New York, NY USA; 776 University Hospital Giessen, Pediatric Hematology and Oncology, Giessen, Germany; 777 Oncologie Sénologie, ICM Institut Régional du Cancer, Montpellier, France; 778 Institute of Clinical Molecular Biology, Christian-Albrechts-University, Kiel, Germany; 779 Institute of Pathology, University of Wuerzburg, Wuerzburg, Germany; 780 Department of Urology, North Bristol NHS Trust, Bristol, UK; 781 SingHealth, Duke-NUS Institute of Precision Medicine, National Heart Centre Singapore, Singapore, Singapore; 782 Department of Computer Science, University of Toronto, Toronto, ON Canada; 783 Bern Center for Precision Medicine, University Hospital of Bern, University of Bern, Bern, Switzerland; 784 Englander Institute for Precision Medicine, Weill Cornell Medicine and New York Presbyterian Hospital, New York, NY USA; 785 Meyer Cancer Center, Weill Cornell Medicine, New York, NY USA; 786 Pathology and Laboratory, Weill Cornell Medical College, New York, NY USA; 787 Vall d’Hebron Institute of Oncology: VHIO, Barcelona, Spain; 788 General and Hepatobiliary-Biliary Surgery, Pancreas Institute, University and Hospital Trust of Verona, Verona, Italy; 789 National Centre for Biological Sciences, Tata Institute of Fundamental Research, Bangalore, India; 790 Indiana University, Bloomington, IN USA; 791 Department of Pathology, GZA-ZNA Hospitals, Antwerp, Belgium; 792 Analytical Biological Services, Inc, Wilmington, DE USA; 793 Sydney Medical School, University of Sydney, Sydney, NSW Australia; 794 cBio Center, Dana-Farber Cancer Institute, Harvard Medical School, Boston, MA USA; 795 Department of Cell Biology, Harvard Medical School, Boston, MA USA; 796 Advanced Centre for Treatment Research and Education in Cancer, Tata Memorial Centre, Navi Mumbai, Maharashtra India; 797 School of Environmental and Life Sciences, Faculty of Science, The University of Newcastle, Ourimbah, NSW Australia; 798 Department of Dermatology, University Hospital of Essen, Essen, Germany; 799 Bioinformatics and Omics Data Analytics, German Cancer Research Center (DKFZ), Heidelberg, Germany; 800 Department of Urology, Charité Universitätsmedizin Berlin, Berlin, Germany; 801 Martini-Clinic, Prostate Cancer Center, University Medical Center Hamburg-Eppendorf, Hamburg, Germany; 802 Department of General Internal Medicine, University of Kiel, Kiel, Germany; 803 German Cancer Consortium (DKTK), Partner site Berlin, Berlin, Germany; 804 Cancer Research Institute, Beth Israel Deaconess Medical Center, Boston, MA USA; 805 University of Pittsburgh, Pittsburgh, PA USA; 806 Department of Ophthalmology and Ocular Genomics Institute, Massachusetts Eye and Ear, Harvard Medical School, Boston, MA USA; 807 Center for Psychiatric Genetics, NorthShore University HealthSystem, Evanston, IL USA; 808 Van Andel Research Institute, Grand Rapids, MI USA; 809 Laboratory of Molecular Medicine, Human Genome Center, Institute of Medical Science, University of Tokyo, Tokyo, Japan; 810 Japan Agency for Medical Research and Development, Tokyo, Japan; 811 Korea University, Seoul, South Korea; 812 Murtha Cancer Center, Walter Reed National Military Medical Center, Bethesda, MD USA; 813 Human Genetics, University of Kiel, Kiel, Germany; 814 Department of Oncologic Pathology, Dana-Farber Cancer Institute, Harvard Medical School, Boston, MA USA; 815 Oregon Health and Science University, Portland, OR USA; 816 Center for RNA Interference and Noncoding RNA, The University of Texas MD Anderson Cancer Center, Houston, TX USA; 817 Department of Experimental Therapeutics, The University of Texas MD Anderson Cancer Center, Houston, TX USA; 818 Department of Gynecologic Oncology and Reproductive Medicine, The University of Texas MD Anderson Cancer Center, Houston, TX USA; 819 University Hospitals Coventry and Warwickshire NHS Trust, Coventry, UK; 820 Department of Radiation Oncology, Radboud University Nijmegen Medical Centre, Nijmegen, GA The Netherlands; 821 Institute for Genomics and Systems Biology, University of Chicago, Chicago, IL USA; 822 Clinic for Hematology and Oncology, St.-Antonius-Hospital, Eschweiler, Germany; 823 Computational and Systems Biology Program, Memorial Sloan Kettering Cancer Center, New York, NY USA; 824 University of Iceland, Reykjavik, Iceland; 825 Division of Computational Genomics and Systems Genetics, German Cancer Research Center (DKFZ), Heidelberg, Germany; 826 Dundee Cancer Centre, Ninewells Hospital, Dundee, UK; 827 Department for Internal Medicine III, University of Ulm and University Hospital of Ulm, Ulm, Germany; 828 Institut Curie, INSERM Unit 830, Paris, France; 829 Department of Gastroenterology and Hepatology, Yokohama City University Graduate School of Medicine, Kanagawa, Japan; 830 Department of Laboratory Medicine, Radboud University Nijmegen Medical Centre, Nijmegen, GA The Netherlands; 831 Division of Cancer Genome Research, German Cancer Research Center (DKFZ), Heidelberg, Germany; 832 Department of General Surgery, Singapore General Hospital, Singapore, Singapore; 833 Cancer Science Institute of Singapore, National University of Singapore, Singapore, Singapore; 834 Department of Medical and Clinical Genetics, Genome-Scale Biology Research Program, University of Helsinki, Helsinki, Finland; 835 East Anglian Medical Genetics Service, Cambridge University Hospitals NHS Foundation Trust, Cambridge, UK; 836 Irving Institute for Cancer Dynamics, Columbia University, New York, NY USA; 837 Institute of Molecular and Cell Biology, Singapore, Singapore; 838 Laboratory of Cancer Epigenome, Division of Medical Science, National Cancer Centre Singapore, Singapore, Singapore; 839 Universite Lyon, INCa-Synergie, Centre Léon Bérard, Lyon, France; 840 Department of Urology, Mayo Clinic, Rochester, MN USA; 841 Royal National Orthopaedic Hospital - Stanmore, Stanmore, Middlesex UK; 842 Department of Biochemistry, Genetics and Immunology, University of Vigo, Vigo, Spain; 843 Giovanni Paolo II / I.R.C.C.S. Cancer Institute, Bari, BA Italy; 844 Neuroblastoma Genomics, German Cancer Research Center (DKFZ), Heidelberg, Germany; 845 Fondazione Policlinico Universitario Gemelli IRCCS, Rome, Italy, Rome, Italy; 846 University of Verona, Verona, Italy; 847 Centre National de Génotypage, CEA - Institute de Génomique, Evry, France; 848 CAPHRI Research School, Maastricht University, Maastricht, ER The Netherlands; 849 Department of Biopathology, Centre Léon Bérard, Lyon, France; 850 Université Claude Bernard Lyon 1, Villeurbanne, France; 851 Core Research for Evolutional Science and Technology (CREST), JST, Tokyo, Japan; 852 Department of Biological Sciences, Laboratory for Medical Science Mathematics, Graduate School of Science, University of Tokyo, Yokohama, Japan; 853 Department of Medical Science Mathematics, Medical Research Institute, Tokyo Medical and Dental University (TMDU), Tokyo, Japan; 854 Cancer Ageing and Somatic Mutation Programme, Wellcome Sanger Institute, Hinxton, UK; 855 University Hospitals Birmingham NHS Foundation Trust, Birmingham, UK; 856 Centre for Cancer Research and Cell Biology, Queen’s University, Belfast, UK; 857 Breast Medical Oncology, The University of Texas MD Anderson Cancer Center, Houston, TX USA; 858 Department of Surgery, Johns Hopkins University School of Medicine, Baltimore, MD USA; 859 Department of Oncology-Pathology, Science for Life Laboratory, Karolinska Institute, Stockholm, Sweden; 860 School of Cancer Sciences, Faculty of Medicine, University of Southampton, Southampton, UK; 861 Department of Gene Technology, Tallinn University of Technology, Tallinn, Estonia; 862 Genetics and Genome Biology Program, SickKids Research Institute, The Hospital for Sick Children, Toronto, ON Canada; 863 Departments of Neurosurgery and Hematology and Medical Oncology, Winship Cancer Institute and School of Medicine, Emory University, Atlanta, GA USA; 864 Department of Clinical and Molecular Medicine, Faculty of Medicine and Health Sciences, Norwegian University of Science and Technology, Trondheim, Norway; 865 Argmix Consulting, North Vancouver, BC Canada; 866 Department of Information Technology, Ghent University, Interuniversitair Micro-Electronica Centrum (IMEC), Ghent, Belgium; 867 Nuffield Department of Surgical Sciences, John Radcliffe Hospital, University of Oxford, Oxford, UK; 868 Institute of Mathematics and Computer Science, University of Latvia, Riga, LV Latvia; 869 Discipline of Pathology, Sydney Medical School, University of Sydney, Sydney, NSW Australia; 870 Department of Applied Mathematics and Theoretical Physics, Centre for Mathematical Sciences, University of Cambridge, Cambridge, UK; 871 Department of Epidemiology and Biostatistics, Memorial Sloan Kettering Cancer Center, New York, NY USA; 872 Department of Statistics, Columbia University, New York, NY USA; 873 Department of Immunology, Genetics and Pathology, Science for Life Laboratory, Uppsala University, Uppsala, Sweden; 874 School of Electronic and Information Engineering, Xi’an Jiaotong University, Xi’an, China; 875 Department of Histopathology, Cambridge University Hospitals NHS Foundation Trust, Cambridge, UK; 876 Oxford NIHR Biomedical Research Centre, University of Oxford, Oxford, UK; 877 Georgia Regents University Cancer Center, Augusta, GA USA; 878 Wythenshawe Hospital, Manchester, UK; 879 Department of Genetics, Washington University School of Medicine, St.Louis, MO USA; 880 Department of Biological Oceanography, Leibniz Institute of Baltic Sea Research, Rostock, Germany; 881 Wellcome Centre for Human Genetics, University of Oxford, Oxford, UK; 882 Department of Molecular and Human Genetics, Baylor College of Medicine, Houston, TX USA; 883 Thoracic Oncology Laboratory, Mayo Clinic, Rochester, MN USA; 884 Institute for Genomic Medicine, Nationwide Children’s Hospital, Columbus, OH USA; 885 Department of Obstetrics and Gynecology, Division of Gynecologic Oncology, Mayo Clinic, Rochester, MN USA; 886 International Institute for Molecular Oncology, Poznań, Poland; 887 Poznan University of Medical Sciences, Poznań, Poland; 888 Genomics and Proteomics Core Facility High Throughput Sequencing Unit, German Cancer Research Center (DKFZ), Heidelberg, Germany; 889 NCCS-VARI Translational Research Laboratory, National Cancer Centre Singapore, Singapore, Singapore; 890 Edison Family Center for Genome Sciences and Systems Biology, Washington University, St. Louis, MO USA; 891 MRC-University of Glasgow Centre for Virus Research, Glasgow, UK; 892 Department of Medical Informatics and Clinical Epidemiology, Division of Bioinformatics and Computational Biology, OHSU Knight Cancer Institute, Oregon Health and Science University, Portland, OR USA; 893 School of Electronic Information and Communications, Huazhong University of Science and Technology, Wuhan, China; 894 Department of Applied Mathematics and Statistics, Johns Hopkins University, Baltimore, MD USA; 895 Department of Cancer Genome Informatics, Graduate School of Medicine, Osaka University, Osaka, Japan; 896 Institute of Computer Science, Heidelberg University, Heidelberg, Germany; 897 School of Mathematics and Statistics, University of Sydney, Sydney, NSW Australia; 898 Ben May Department for Cancer Research, University of Chicago, Chicago, IL USA; 899 Department of Human Genetics, University of Chicago, Chicago, IL USA; 900 Tri-Institutional PhD Program in Computational Biology and Medicine, Weill Cornell Medicine, New York, NY USA; 901 The First Affiliated Hospital, Xi’an Jiaotong University, Xi’an, China; 902 Department of Medicine and Therapeutics, The Chinese University of Hong Kong, Shatin, NT, Hong Kong China; 903 Department of Biostatistics, The University of Texas MD Anderson Cancer Center, Houston, TX USA; 904 Duke-NUS Medical School, Singapore, Singapore; 905 Department of Surgery, Ruijin Hospital, Shanghai Jiaotong University School of Medicine, Shanghai, China; 906 School of Computing Science, University of Glasgow, Glasgow, UK; 907 Division of Orthopaedic Surgery, Oslo University Hospital, Oslo, Norway; 908 Eastern Clinical School, Monash University, Melbourne, VIC Australia; 909 Epworth HealthCare, Richmond, VIC Australia; 910 Department of Biostatistics and Computational Biology, Dana-Farber Cancer Institute and Harvard Medical School, Boston, MA USA; 911 Department of Biomedical Informatics, College of Medicine, The Ohio State University, Columbus, OH USA; 912 The Ohio State University Comprehensive Cancer Center (OSUCCC – James), Columbus, OH USA; 913 The University of Texas School of Biomedical Informatics (SBMI) at Houston, Houston, TX USA; 914 Department of Biostatistics, University of North Carolina at Chapel Hill, Chapel Hill, NC USA; 915 Department of Biochemistry and Molecular Genetics, Feinberg School of Medicine, Northwestern University, Chicago, IL USA; 916 Faculty of Medicine and Health, University of Sydney, Sydney, NSW Australia; 917 Department of Pathology, Erasmus Medical Center Rotterdam, Rotterdam, GD The Netherlands; 918 Division of Molecular Carcinogenesis, The Netherlands Cancer Institute, Amsterdam, CX The Netherlands; 919 Institute of Molecular Life Sciences and Swiss Institute of Bioinformatics, University of Zurich, Zurich, Switzerland

**Keywords:** Cancer genomics, Data integration, Gene ontology

## Abstract

Multi-omics datasets represent distinct aspects of the central dogma of molecular biology. Such high-dimensional molecular profiles pose challenges to data interpretation and hypothesis generation. ActivePathways is an integrative method that discovers significantly enriched pathways across multiple datasets using statistical data fusion, rationalizes contributing evidence and highlights associated genes. As part of the ICGC/TCGA Pan-Cancer Analysis of Whole Genomes (PCAWG) Consortium, which aggregated whole genome sequencing data from 2658 cancers across 38 tumor types, we integrated genes with coding and non-coding mutations and revealed frequently mutated pathways and additional cancer genes with infrequent mutations. We also analyzed prognostic molecular pathways by integrating genomic and transcriptomic features of 1780 breast cancers and highlighted associations with immune response and anti-apoptotic signaling. Integration of ChIP-seq and RNA-seq data for master regulators of the Hippo pathway across normal human tissues identified processes of tissue regeneration and stem cell regulation. ActivePathways is a versatile method that improves systems-level understanding of cellular organization in health and disease through integration of multiple molecular datasets and pathway annotations.

## Abstract (web summary)

Multi-omics datasets pose major challenges to data interpretation and hypothesis generation owing to their high-dimensional molecular profiles. Here, the authors develop ActivePathways method, which uses data fusion techniques for integrative pathway analysis of multi-omics data and candidate gene discovery.

## Introduction

Pathway enrichment analysis is an essential step for interpreting high-throughput (omics) data that uses current knowledge of genes and biological processes. A common application determines statistical enrichment of molecular pathways, biological processes and other functional annotations in long lists of candidate genes^1^. Genomic, transcriptomic, proteomic and epigenomic experiments emphasize complementary aspects of underlying biology and are best analyzed integratively, as is now routinely done in large-scale projects such as The Cancer Genome Atlas (TCGA)^2^, Clinical Proteome Tumor Analysis Consortium (CPTAC), International Cancer Genome Consortium (ICGC)^3,4^, Genotype-Tissue Expression (GTEx)^5^, and others^6^. Thus, simultaneous analysis of multiple candidate gene lists for characteristic pathways is increasingly needed.

Numerous approaches are available for interpreting single gene lists. For example, the GSEA algorithm can detect upregulated and downregulated pathways in gene expression datasets^7^. Web-based methods such as Panther^8^, ToppCluster^9^, and g:Profiler^10^ detect significantly enriched pathways amongst ranked or unranked gene lists and are generally applicable to genes and proteins from various analyses. Some approaches allow analysis of multiple input gene lists however these primarily rely on visualization rather than data integration to evaluate the contribution of distinct gene lists towards each detected pathway^9,10^. Finally, no methods are available for unified pathway analysis of coding and non-coding mutations from whole-genome sequencing (WGS) data, or integrating these with other types of DNA aberrations such as copy number changes and balanced genomic rearrangements.

Cancer genomes are characterized by multiple classes of mutations, including single nucleotide variants (SNVs), small insertions–deletions (indels), copy number alterations, and translocations. These affect a small number of frequently mutated pan-cancer driver genes such as *TP53*, less-frequent and tissue-specific genes such as *SPOP* in prostate cancer, and numerous infrequently mutated genes. The majority of currently known driver mutations of SNVs and indels affect protein-coding sequence^11^ and only few high-confidence non-coding drivers have been found, such as the mutation hotspots in the *TERT* promoter^12^. Discovery of coding and non-coding driver mutations is a major goal of large cancer whole genome sequencing efforts. The PCAWG Consortium aggregated whole genome sequencing data from 2658 cancers across 38 tumor types generated by the ICGC and TCGA projects. These sequencing data were re-analysed with standardized, high-accuracy pipelines to align to the human genome (reference build hs37d5) and identify germline variants and somatically acquired mutations, as described in the PCAWG marker paper^4^. A consensus analysis of variant calls in PCAWG tumors generated a high-confidence catalog of driver mutations in protein-coding driver genes (CDS) and non-coding regions of 5′ and 3′ untranslated elements (UTRs), promoters and enhancers^13^. A consensus pathway and network analysis of PCAWG driver mutations used knowledge of molecular pathways and gene interaction networks as priors to further discover infrequent candidate driver variants including those in the non-coding genome^14^.

Here we report the development of the ActivePathways method that uses data fusion techniques to address the challenge of integrative pathway analysis of multi-omics data. It detects significantly enriched pathways across multiple datasets, including those pathways that are not apparent in any individual dataset. We present several analyses to demonstrate this method. First, we integrate cancer driver genes with coding and non-coding mutations predicted using the PCAWG dataset^13^ and reveal numerous processes and additional genes with frequent coding and non-coding mutations. Second, we integrate patient clinical information with transcriptomic and copy number alterations in breast cancers of the METABRIC project^15^ to discover prognostic pathways and processes in breast cancer subtypes. Third, we integrate transcriptomic data of normal tissues of the GTEx project^5^ with ChIP-seq data to infer gene regulatory networks and biological processes downstream of the Hippo pathway of tissue growth control and regeneration. Thus ActivePathways is a versatile method for combining diverse multiomics datasets.

## Results

### Multi-omics pathway enrichment analysis with ActivePathways

**Fig. 1:**
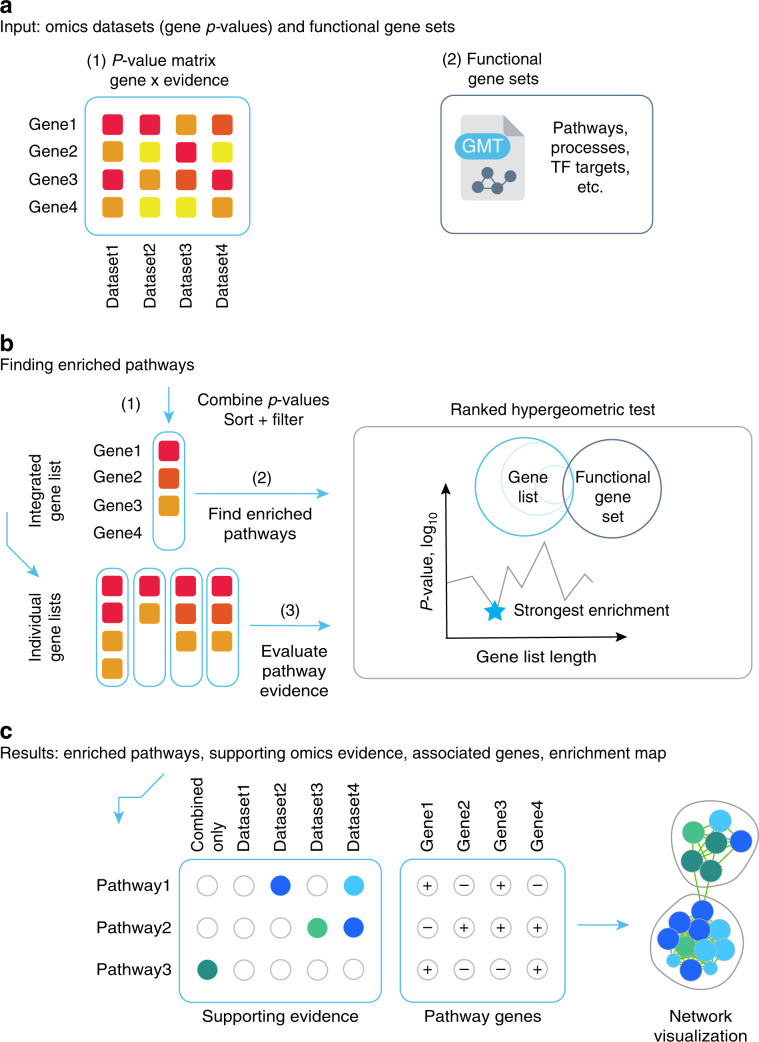
Method overview. **a** ActivePathways requires as input (1) a matrix of gene *P*-values for different omics datasets, and (2) a collection of gene sets corresponding to biological pathways and processes. **b** In step (1), gene *P*-values are merged using the Brown procedure and filtered to produce an integrated gene list that combines evidence from datasets and is ranked by decreasing significance with a lenient threshold. In step (2), pathway enrichment analysis is conducted on the integrated gene list using the ranked hypergeometric test that determines the optimal level of enrichment in the ranked gene sub-list for every pathway. In step (3), separate gene lists are compiled from individual input datasets and analyzed for pathway enrichment using the ranked hypergeometric test, to find supporting evidence for each pathway from the integrative analysis. **c** ActivePathways provides a list of enriched pathways in the integrated gene list, the associated genes with significant Brown *P*-values, and annotations of evidence supporting each pathway. Results of ActivePathways are visualized as enrichment maps where nodes correspond to pathways and pathways with many shared genes are connected into networks representing broader biological themes.

ActivePathways is a simple three-step method that extends our earlier work^10^ (Fig. 1). It requires two input datasets. The first input is a table of *P*-values with genes listed in rows and evidence from distinct datasets listed in columns. The columns can include *P*-values of differential gene expression, gene essentiality, mutation or copy number alteration burden and many others that are derived using platform-specific quantification methods. The second input is a collection of gene sets that represents collective knowledge of gene function and interactions we refer to as pathways. The most common analysis utilizes biological processes from gene ontology^16^ (GO) and molecular pathways from the Reactome database^17^. Depending on the hypothesis, these data may also include many other types of gene sets such as targets of transcription factors or microRNAs.

In the first step of ActivePathways, we derive an integrated gene list that for each input gene aggregates significance from multiple omics datasets. The integrated gene list is compiled by a fusion of gene significance from different omics datasets (i.e., evidence) using the Brown’s extension^18^ of the Fisher’s combined probability test. The Brown’s method considers dependencies between datasets and thus provides more conservative estimates of significance for genes that are supported by multiple similar omics datasets. The integrated input gene list is then ranked by decreasing significance and filtered using a lenient cut-off, designed to capture additional candidate genes with sub-significant signals while discarding the bulk of insignificant genes (unadjusted Brown *P*_gene_ < 0.1). In the second step, a pathway enrichment analysis is conducted on the integrated gene using a ranked hypergeometric test^10^ and a collection of gene sets (i.e., biological processes, molecular pathways, and other gene annotations). The ranked hypergeometric test is designed to capture smaller pathways tightly associated with few top-ranking genes and also broader processes associated with larger subsets of input genes. The family-wise multiple testing correction method by Holm^19^ is then applied across tested pathways to select the pathways significantly enriched in the integrated gene list (*Q*_pathway_ < 0.05). In the third step, we perform a similar analysis on the gene lists of individual omics datasets separately to determine the omics evidence supporting the integrative pathway analysis results determined in step 2. Importantly, the third step also highlights pathways that are only found through data integration and are not apparent in any single omics dataset separately, providing the added value of integrated analysis. Finally, the method provides input files for Enrichment Map^20^ for visualizing resulting pathways with the corresponding omics evidence.

### Integrating coding and non-coding drivers in 2658 cancer genomes

**Fig. 2:**
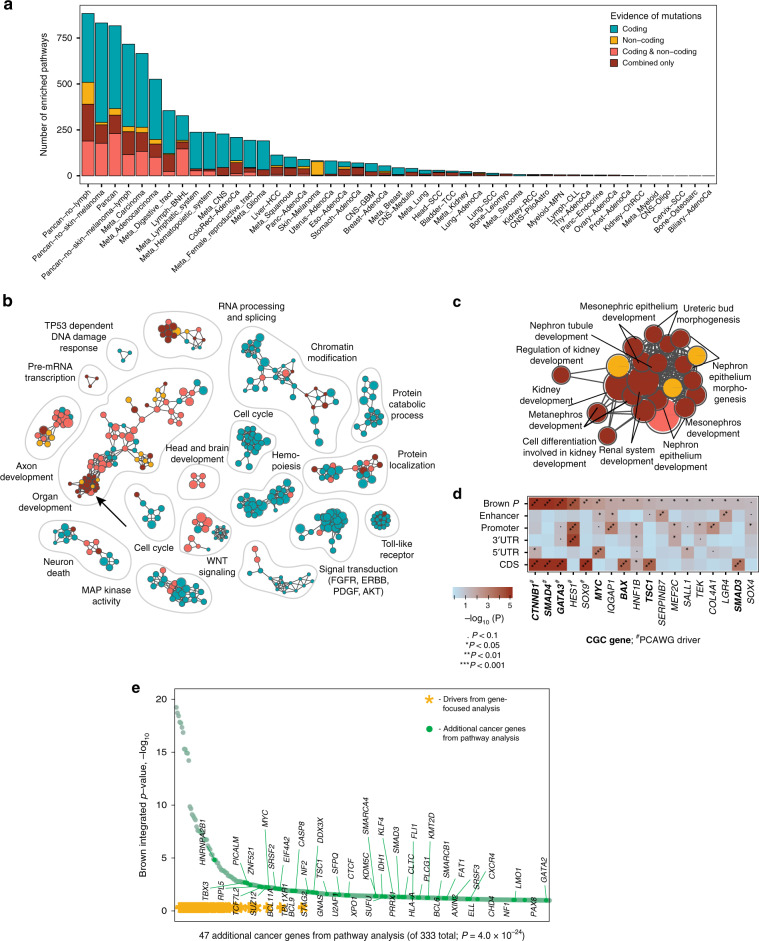
Pathway enrichment analysis of cancer driver genes with ActivePathways. **a** Bar plot shows number of significantly enriched pathways (*Q* < 0.05) among predicted driver genes with coding and non-coding mutations in the PCAWG dataset. The majority of pathways detected by ActivePathways are supported by protein-coding mutations, as expected (dark green bars), while non-coding mutations (orange, red) reveal additional pathways. Pathways shown in dark red are found only in the integrated gene list of coding and non-coding mutations but not in gene lists of individual mutation scores. **b** Enrichment map shows pathways enriched in frequently mutated genes in the adenocarcinoma cohort of 1773 tumors. Nodes in the network represent pathways and similar pathways with many common genes are connected. Groups of similar pathways are indicated. Nodes are colored by supporting evidence from coding and non-coding cancer mutations. Arrow indicates kidney developmental processes. **c** The group of enriched kidney developmental processes is apparent from integrated evidence of coding and non-coding mutations but is not found among coding or non-coding candidate genes separately. **d** Heatmap shows *P*-values of driver genes involved in kidney developmental processes, including driver genes found in the driver analysis (indicated with #) and additional genes only found in the pathway analysis. Top row shows merged *P*-values from the Brown procedure. Genes listed in the Cancer Gene Census (CGC) database are indicated in boldface letters. **e** Pathway analysis recovers most genes of the driver list from PCAWG (orange asterisks), as well as additional infrequently mutated genes apparent due to their pathway associations. Additional known cancer genes detected in the pathway analysis are listed (green dots) and occur more frequently than expected from chance alone.

We performed an integrative pathway analysis of driver genes predicted in the PCAWG project based on somatic SNVs and indels. This analysis comprised 29 cancer patients cohorts of histological tumor types and 18 meta-cohorts combining multiple types of tumors, with 47 cohorts in total (Supplementary Table 1). ActivePathways identified at least one significantly enriched process or pathway in 89% of these cohorts (42/47, *Q*_pathway_ < 0.05, ranked hypergeometric test) (Fig. 2a). We analyzed the evidence supporting predictions of enriched pathways: most cohorts showed enrichments in pathways supported by protein-coding mutations in genes (37/47 or 79%). This serves as a positive control since the majority of currently-known cancer driver genes have frequent protein-coding mutations.

Non-coding mutations in genes also contributed broadly to the discovery of frequently mutated biological processes and pathways: 24/47 cohorts (51%) showed significantly enriched pathways that were apparent when only analyzing non-coding driver scores corresponding to UTRs, promoters or enhancers. The majority of PCAWG tumor cohorts (41/47 or 87%) revealed some frequently mutated pathways that were apparent when integrating coding and non-coding mutations however remained undetected when considering either coding or non-coding mutations separately, emphasizing the value of our integrative approach. As expected, cohorts with more patient tumor samples generated more significantly enriched pathways (Spearman *ρ* = 0.74, *P* = 2.3 × 10^−9^; Supplementary Fig. 1), suggesting that larger datasets are better powered to distinguish rarely mutated genes involved in biological pathways and processes. Discovery of pathways enriched in non-coding mutations suggests that our integrative pathway analysis is an attractive strategy for illuminating the dark matter of the non-coding cancer genome.

We studied the adenocarcinoma cohort of 1773 samples of 16 tumor types. Integrative pathway analysis highlighted 432 genes that were significantly enriched in 526 pathways (*Q*_pathway_ < 0.05) (Fig. 2b). As expected, the majority of pathways were only supported by genes with frequent coding mutations (328/526 or 62%). However, an additional set of 101 pathways (19%) was supported by both coding and non-coding gene mutations, 72 pathways (14%) were only apparent in the integrated analysis of both coding and non-coding mutations, and 25 (5%) were only enriched in non-coding mutations. Accumulation of these individually infrequent non-coding mutations into relevant pathways and processes is apparent in our integrative analysis and remains undetected in a gene-focused analysis.

The major biological themes with frequent protein-coding mutations included hallmark cancer processes such as ‘apoptotic signaling’ (24 genes; *Q*_pathway_ = 4.3 × 10^−5^) and ‘mitotic cell cycle’ (8 genes; *Q*_pathway_ = 0.0026), and additional biological processes such as chromatin modification and RNA splicing that are increasingly recognized in cancer biology. Thus, ActivePathways captures the expected cancer pathways enriched in driver genes with protein-coding mutations as positive controls. In contrast to these solely protein-coding driver associations, a large group of developmental processes and signal transduction pathways was detected as enriched in both coding and non-coding mutations in genes; for example ‘embryo development process’ was supported by mutations in exons, 3′UTRs and gene promoters (68 genes; *Q*_pathway_ = 2.9 × 10^−12^), while the Reactome pathway ‘repression of WNT target genes’ was only apparent in the integrated analysis of coding and non-coding mutations but not in either dataset alone (5 genes, *Q*_pathway_ = 0.016). In summary, these data show that ActivePathways is a sensitive approach for integrating multi-omics signals such as coding and non-coding mutations, interpreting supporting omics evidence, and finding additional functional associations that are not apparent in any single input dataset.

### Pathway-based prioritization of rarely mutated cancer genes

Pathway analysis can identify candidate genes that would otherwise remain undetected in gene-based analyses. ActivePathways enhances such discovery by integrating signals across multiple datasets. In the pathway analysis of coding and non-coding mutations in PCAWG, we focused on a group of processes involved in kidney development that were exclusively detected through the integration of coding and non-coding mutations (Fig. 2c, d). ActivePathways found 18 genes involved in these processes, only five of which were predicted as driver genes in the consensus driver analysis of the PCAWG project^13^. Additional known cancer genes included the oncogene *MYC* with 13 patients with 3′UTR mutations (driver *P*-value *P*_UTR3_ = 4.8 × 10^−4^), the transcription factor *SMAD3* of the TGF-β pathway with 14 patients with protein-coding mutations (*P*_CDS_ = 4.0 × 10^−4^) and the growth inhibitory tumor suppressor gene *TSC1* with 23 patients with protein-coding mutations (*P*_CDS_ = 1.4 × 10^−4^) as well as candidate cancer genes such as *IQGAP1* with ten patients with promoter mutations (*P*_promoter_ = 8.2 × 10^−4^) that encodes a signaling protein involved in cell motility and morphology. The additional genes remained below the FDR-adjusted significance cut-off in the gene-focused consensus driver analysis (*Q* = 0.17–0.62), however were found by ActivePathways due to their associations with kidney development. Thus ActivePathways can exploit functional gene annotations and multiple omics signals to find further candidate genes that remain undetected in gene-focused analyses.

We evaluated the effects of our data integration strategy and examined all 333 pathway-associated candidate genes detected in the adenocarcinoma cohort (Fig. 2e). As expected, these included a considerable proportion of known cancer genes. First, as positive controls we found 60/64 significantly mutated genes that were also identified in the PCAWG consensus driver analysis^13^. In addition, we found a set of 47/333 known cancer genes annotated in the COSMIC Cancer Gene Census database^11^ that were not detected in the driver analysis, significantly more than expected by chance alone (seven genes expected, Fisher’s exact *P* = 4.0 × 10^−24^). Those included well-known cancer driver genes *MYC, IDH1, NF1*, and *BCL9*. ActivePathways was able to detect these additional genes for several reasons. First, the integrated gene list was filtered using a lenient statistical cut-off in ActivePathways (*P*_gene_ < 0.1) that allowed a long tail of 273/333 genes with less-frequent mutations to be detected through pathway associations. Second, certain genes were upgraded through the data fusion procedure as a single stronger *P*-value per gene was derived by combining multiple weaker *P*-values corresponding to the coding regions, promoters, UTRs, enhancers of the gene. This affected 17/333 pathway-associated genes including six known cancer genes (*HNRNPA2B1*, *STAG2*, *TCF7L2*, *SUZ12*, *CLTC*, and *ZNF521*), Thus, the integration procedure prioritized specific pathway-related genes compared to their original rankings in individual mutation datasets. However, the majority of genes showed reduced significance after the fusion and were excluded from the pathway analysis: 3543 genes had at least one significant *P*-value prior to data fusion (*P*_gene_ < 0.1) while 88% of these (3112) were considered insignificant following the Brown *P*-value combination step. In contrast, the majority of pathway-associated genes (220/333) showed improved rankings in the integrated gene list compared to their original rankings in individual input datasets. This formal combination of *P*-values across omics datasets is therefore more conservative than a naïve approach of selecting a top *P*-value for every gene. Thus, ActivePathways finds additional candidate genes that remain undetected in gene-by-gene analyses and are highlighted due to their multiple omics signals in pathways.

### Integrating prognostic CNA and mRNA signals in breast cancer

To demonstrate an integrative analysis of patient clinical information with multiple types of omics data, we studied the pathways and processes associated with patient prognosis in breast cancer. We leveraged the METABRIC dataset^15^ of 1780 breast cancer samples drawn from all four subtypes (HER2-enriched, basal-like, luminal-A, luminal-B) and evaluated all genes using three types of prognostic evidence. mRNA abundance profiles were deconvolved between mRNA abundance levels in tumor cells (TC) and tumor-adjacent cells (TAC) using the ISOpure algorithm^21,22^. mRNA values were associated with patient survival using median dichotomization and log-rank tests. Gene copy number alterations (CNA) were included as the third type of evidence and associated with patient survival using log-rank tests.

**Fig. 3:**
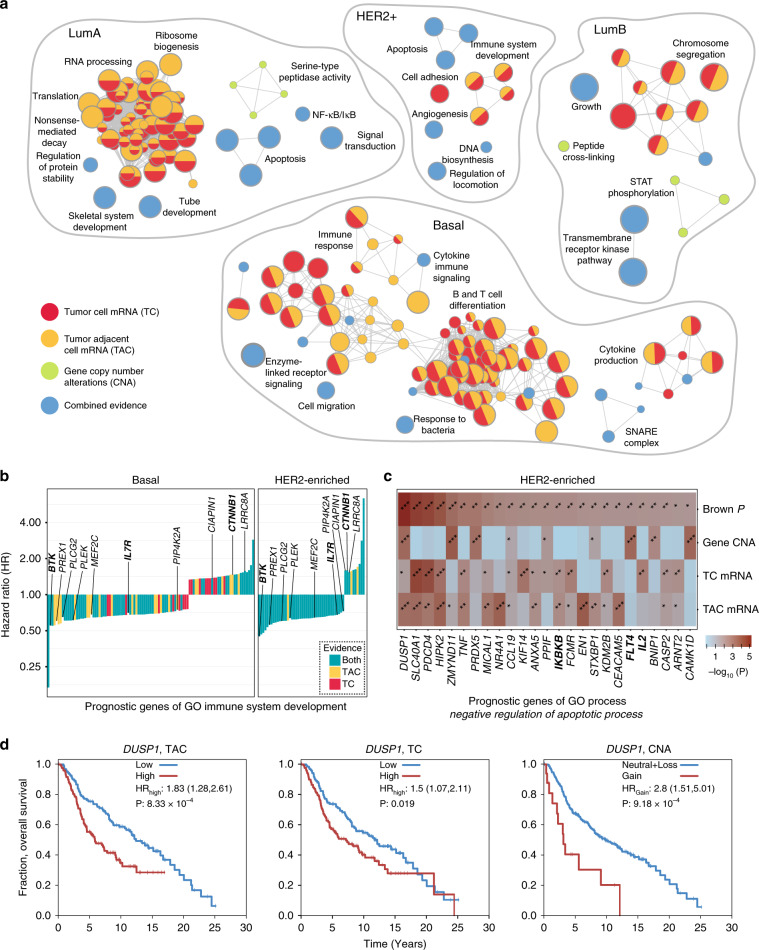
Prognosis-associated pathways in four molecular subtypes of breast cancer. **a** Enrichment maps of prognostic pathways and processes were found in an integrative analysis of mRNA abundance in tumor cells (TC), tumor-adjacent cells (TAC), and gene copy number alterations (CNA) of the METABRIC dataset. Multicolored nodes indicate pathways that were prognostic according to several types of molecular evidence. Blue nodes indicate pathways that were only apparent through merging of molecular signals. **b** Hazard ratios (HR) of prognostic genes related to immune system development in basal and HER2-enriched subtypes of breast cancer. Strongest HR value of TC, TAC is shown. Genes commonly found in basal and HER2-enriched tumors are shown. Known cancer genes are shown in boldface. **c** Heatmap shows genes, corresponding log-rank *P*-values, and merged Brown *P*-values related to the GO process ‘negative regulation of apoptotic process’ that was found by integrating prognostic omics data in HER2-enriched breast cancer. **d** Kaplan–Meier plots show the strongest prognostic signal related to apoptotic signaling, the phosphatase *DUSP1* that significantly associates with reduced patient survival through increased tumor-adjacent mRNA level (left), increased tumor mRNA level (center) and gene copy number amplification (right). Log-rank *P*-values are shown.

ActivePathways identified 192 significantly enriched GO biological processes and Reactome pathways across the four subtypes of breast cancer, of which nine pathways were enriched in multiple cancer subtypes and 33 pathways were only apparent through the integrative pathway analysis but not in any of the CNA or mRNA datasets alone. The major findings enriched in prognostic signatures in breast cancer subtypes involved the processes and pathways of immune response, apoptosis, ribosome biogenesis and chromosome segregation (Fig. 3a).

Immune activity was associated with prognostic genes in basal-like and HER2-enriched breast cancers with significant enrichment of GO processes such as ‘immune system development’ (*Q*_basal_ = 3.0 × 10^−4^, 113 genes; *Q*_*HER2*_ = 0.035, 61 genes; ranked hypergeometric test) and ‘lymphocyte differentiation’ (*Q*_*HER2*_ = 6.8 × 10^−4^, 46 genes; *Q*_basal_ = 8.4 × 10^−4^, 45 genes). The majority of related genes were associated with improved patient prognosis upon increased mRNA abundance in tumor cells or tumor-adjacent cells, comprising 50/61 genes in the HER2-enriched subtype and 78/113 genes in the basal subtype (Fig. 3b). Interestingly, only a minority of these genes (10) were significant in both of the two breast cancer subtypes, suggesting different modes of immune activity in subtypes and emphasizing the power of our pathway-based approach. Basal-like breast cancers were associated with additional 67 terms involving immune response and blood cells, however no immune-related terms were enriched for luminal subtypes of breast cancers. Prognostic features of immune-related genes in HER2-enriched and basal-like breast cancers are well known^23,24^. Our pathway-based findings indicate that immune activity in breast tumor cells and in the surrounding microenvironment negatively affects tumor progression and improves prognosis.

Apoptosis was associated with patient prognosis in HER2-enriched and luminal-A breast cancers through enriched GO processes such as ‘negative regulation of apoptotic process’ (*Q*_*HER2*_ = 0.030, 122 genes; *Q*_luminalA_ = 0.015, 228 genes) and ‘programmed cell death’ (*Q*_*HER2*_ = 0.015, 125 genes; *Q*_luminalA_ = 0.016, 231 genes) (Fig. 3c). Interestingly, anti-apoptotic pathways were only detected in the integrative analysis and not in genomic and transcriptomic gene signatures separately. Among the genes negatively regulating apoptosis, *DUSP1* provided the strongest prognostic signal in HER2-enriched breast cancers. This was apparent in the molecular stratification of samples by mRNA of tumor cells (log-rank *P*_TC_ = 0.019, HR = 1.5) and tumor-adjacent cells (*P*_TAC_ = 8.3 × 10^−4^, HR = 1.8) as well as gene copy number amplifications (*P*_CNA_ = 9.8 × 10^−4^, HR = 2.8) (Fig. 3d). *DUSP1* encodes a phosphatase signaling protein of the MAPK pathway that is over-expressed in malignant breast cancer cells and inhibits apoptotic signaling^25^. *HER2* over-expression is known to suppress apoptosis in breast cancer^26^. Anti-apoptotic signaling is a hallmark of cancer and expectedly associated with worse patient prognosis.

ActivePathways also identified prognostic pathway associations that were only apparent in single subtypes of breast cancer. For example, the prognostic genes for luminal-B subtype were enriched in processes related to ‘chromosome segregation’ (*Q*_luminalB_ = 0.017, 41 genes) that have been associated with worse outcome in breast cancer^27^. As another example, luminal-A breast cancers were associated with prognosis in ribosomal and RNA processing genes, such as ‘ribosome biogenesis’ (*Q*_luminalA_ = 6.9 × 10^−10^, 60 genes) and ‘rRNA metabolic process’ (*Q*_luminalA_ = 1.8 × 10^−13^, 64 genes). Although not described specifically in the luminal-A subtype, ribosomal mRNA abundance has been shown to be prognostic in breast cancer as a marker of cell proliferation^28,29^. In summary, ActivePathways can be used for integrating clinical data with multiomics information of molecular alterations. Such analyses can provide leads for functional studies and biomarker development.

### Interpreting co-expressed and DNA-bound targets of Hippo TFs

To demonstrate the use of ActivePathways for studying gene regulation, we analyzed transcriptomes of non-cancerous human tissues from the GTEx project^5^. We focused on the Hippo signaling pathway involved in organ size control, tissue homeostasis and cancer^30,31^ and studied regulatory networks downstream of the two transcription factors (TFs) YAP and TAZ (encoded by *YAP1* and *WWTR1*). YAP and TAZ are evolutionarily conserved master regulators of Hippo signaling in mammals that respond to intracellular and extracellular signals of cell–cell interactions, cell polarity, mechanical cues, G protein-coupled receptor signaling, and cellular energy status^30,31^.

We performed an integrative pathway enrichment analysis of transcriptomics and epigenomics data of the two master regulators of the Hippo pathway. First, we predicted transcriptional target genes for YAP and TAZ (1898 and 1319 genes, respectively), using co-expression analysis and robust rank aggregration^32^ over 9642 transcriptomes of 40 tissue types in GTEx (*Q* < 0.05). Second, we studied the set of 2356 target genes of YAP that have DNA-binding sites at gene promoters derived from a YAP ChIP-seq study^33^ reanalyzed in the ReMap database^34^ (*Q* < 0.05). The three gene lists with corresponding significance values were used as input to ActivePathways for the integrative analysis.

**Fig. 4:**
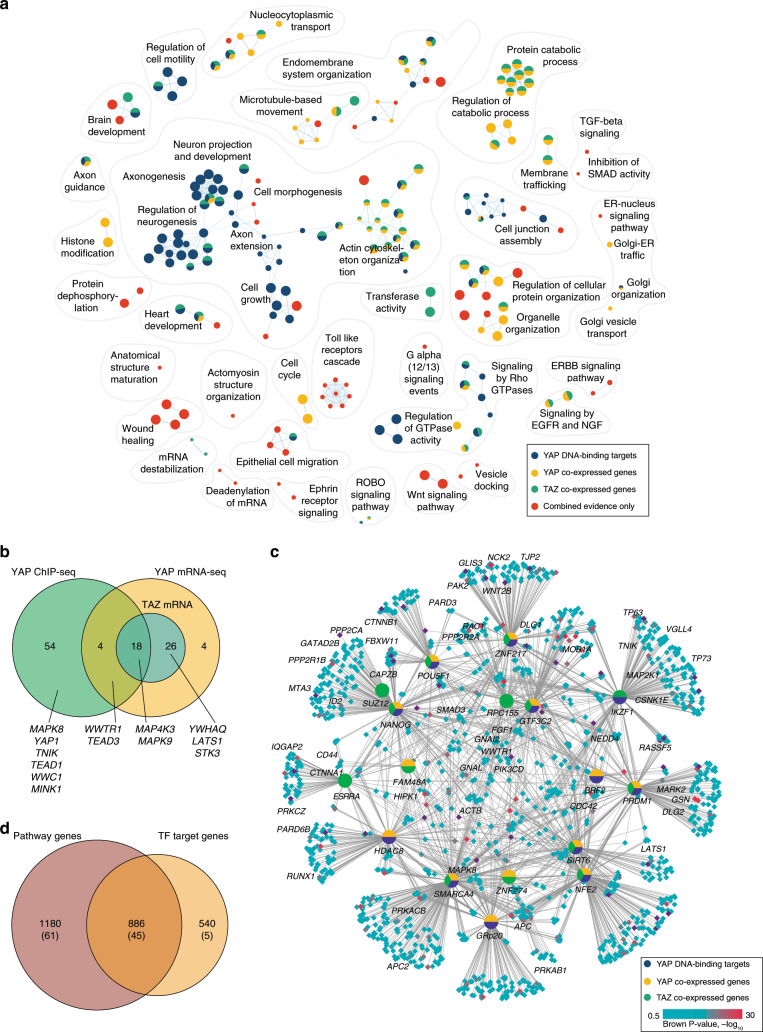
Integrative pathway enrichment analysis of Hippo target genes across human tissues. **a** Enrichment map of GO processes and Reactome pathways enriched in the target genes of transcriptional regulators YAP and TAZ of the Hippo pathway. Co-expressed target genes of YAP and TAZ across normal human tissues of the GTEx dataset (pathways are shown in green and yellow, respectively) and DNA-binding target genes of YAP from ChIP-seq experiments (shown in blue) were analyzed. Pathways only found through the integration of ChIP-seq and RNA-seq data are shown in red. **b** Euler diagram shows 106 Hippo-related genes that were significantly enriched in the detected pathways and supported by a combination of signals in RNA-seq and ChIP-seq datasets. Core Hippo genes detected in the analysis are listed. **c** Regulatory network of 17 TFs and 1,426 target genes detected in the ActivePathways analysis of gene sets representing transcription factor target genes. Transcription factors with enriched target genes in the YAP/TAZ regulome are shown in multi-colored circles. Target genes are colored by increasing statistical significance (turquoise to red). **d** Integrative analysis of pathways and GO processes is complementary to the analysis of transcription factor targets. Euler diagram shows total number of pathway-associated identified in the analysis of GO and Reactome terms (left) and TF target genes from ENCODE (right). Numbers of Hippo-related genes are shown in brackets.

Integrative analysis of transcriptional and DNA-binding target genes of YAP/TAZ resulted in 225 significantly enriched GO processes and Reactome pathways (*Q*_pathway_ < 0.05) (Fig. 4a). The resulting pathways are expected in the context of Hippo signaling and included development and morphogenesis, cell motility, organization of actin cytoskeleton and cell–cell junctions, signal transduction pathways such as EGFR, Wnt, Robo, TGF-beta, rho GTPase, and others. ActivePathways highlighted 2066 genes in these pathways. We examined known members of the Hippo pathway and found 13 of 32 genes among pathway-associated genes (*WWTR1*, *LATS1*, *WWC1*, *YWHAQ*, *YAP1*, *MAPK8*, *MAPK9*, *STK3*, *TNIK*, *TEAD3*, *MAP4K3*, *MINK1*, *TEAD1*), more than expected from chance alone (four genes expected, *P* = 3.6 × 10^−5^, Fisher’s exact test). We also compiled an extended list of 106 Hippo-related genes^30,31,35^ and confirmed that these were enriched in pathway-associated genes (36/308 genes expected, *P* = 9.1 × 10^−26^, Fisher’s exact test) (Fig. 4b). A large fraction of pathways (55 or 24%) was only identified in the integrated analysis and not in any input dataset alone, underlining the advantage of detecting significantly enriched pathways across multiple complementary omics datasets. Similarly, Hippo-related genes were either supported by ChIP-seq data alone (54 genes), mRNA data alone (30 genes), or both by mRNA and ChIP-seq data (22 genes), concordant with the notion that RNA-seq and ChIP-seq show limited agreement regarding TF target genes and provide complementary insights into gene regulation. Thus, our integrative analysis of co-expression networks and TF–DNA interactions of Hippo master regulators expectedly converges on Hippo-related genes and pathways.

In addition to GO terms and pathways, ActivePathways can be used to interpret omics data with other classes of functional gene sets such as TF target genes. To further elucidate gene regulatory networks downstream of YAP/TAZ, we considered potential enrichments of DNA-binding target genes of 161 TFs profiled in ChIP-seq studies of the ENCODE project^36^. We found a regulatory network of 17 TFs and 1426 target genes enriched in the YAP/TAZ regulome (*Q* < 0.05, ranked hypergeometric test) (Fig. 4c). These included the DNA-binding targets of the master regulators of pluripotent stem cells^37^ NANOG (208/774 target genes, *Q* = 1.3 × 10^−6^) and POU5F1 (107/406 target genes, *Q* = 7.3 × 10^−11^). This finding is in agreement with the role of Hippo pathway activity in stem cell regulation^38^. The regulatory network was significantly enriched in 50 Hippo-related genes (25 expected, *P* = 1.2 × 10^−7^, Fisher’s exact test) and six core Hippo genes (two expected*, P* = 0.030*; WWTR1*, *VGLL4*, *TNIK*, *MAPK8*, *MOB1A*, *LATS1*), similarly to the pathway-based analysis above. However, the two analyses revealed distinct genes: 886 genes were commonly found by both analyses, 1180 genes were found only in the pathway-based analysis, and 540 genes were found only in the TF-based analysis (Fig. 4d). Thus, integrative enrichment analysis of TF target genes provides complementary information to GO terms and pathways. In summary, this analysis highlights genes and pathways related to Hippo signaling in human tissues and demonstrates the use of ActivePathways for studying gene regulatory networks across complementary omics datasets and technology platforms.

### Evaluating the robustness and sensitivity of ActivePathways

**Fig. 5:**
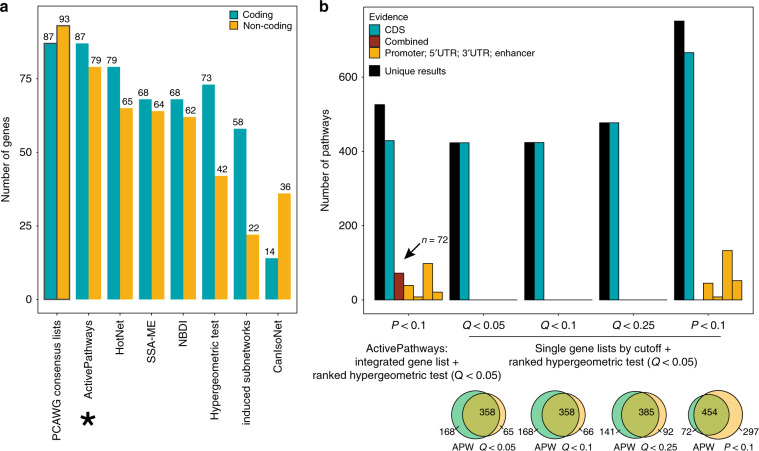
Benchmarking of ActivePathways. **a** Comparison of ActivePathways with six additional pathway and network analysis methods used in the PCAWG pathway and network consensus analysis. ActivePathways best recovers the consensus lists of pathway-implicated driver genes with coding and non-coding mutations (indicated by asterisk). The consensus lists are shown in the leftmost bars of the plot and have been compiled through a majority vote of the seven methods in the PCAWG pathway and network analysis working group. **b** Comparison of ActivePathways (leftmost bars) and common pathway enrichment analysis using multiple significance cut-offs of PCAWG gene lists with protein-coding and non-coding mutations. ActivePathways shows increased sensitivity of pathway analysis even at the most lenient gene list significance cut-offs and recovers additional pathways only detected through integration of multiple datasets (dark red).

We carefully benchmarked ActivePathways using the dataset of cancer driver genes predicted by PCAWG^13^. First, we compared the performance of ActivePathways with six methods used in the PCAWG pathway and network analysis working group^14^ (Hierarchical HotNet^39,40^, SSA−ME^41^, NBDI^42^, induced subnetwork analysis^40^, CanIsoNet^43^, hypergeometric test). These diverse methods used molecular interaction networks, functional gene sets and/or transcriptomics data to analyze the PCAWG pan-cancer dataset of predicted cancer driver genes. A subsequent consensus analysis defined pathway-implicated driver gene lists with protein-coding and non-coding mutations, based on a majority vote of the pathway and network analysis methods^14^. ActivePathways recovered these consensus gene lists with the highest accuracy: 100% of coding driver genes (87/87) and 85% of non-coding candidate genes (79/93) were detected (Fig. 5a). Thus ActivePathways agreed the most with the ensemble of several other methods in prioritizing known and candidate cancer driver genes using pathway and network context.

We also compared the performance of ActivePathways with a standard approach of pathway enrichment analysis that considers a single statistically-filtered gene list using a ranked hypergeometric test^1,10^ (Fig. 5b). To this end, we analyzed individual gene lists of protein-coding and non-coding drivers of the PCAWG adenocarcinoma cohort using multiple gene selection thresholds (*Q* < 0.05, *Q* < 0.1, *Q* < 0.25, *P* < 0.1). ActivePathways showed increased sensitivity of pathway enrichment analysis compared to the standard approach, in particular for pathways involving non-coding mutations that were not promimently represented in any single gene lists. Even compared to the analysis employing the most lenient gene selection filter (*P* < 0.1), ActivePathways identified 72 additional pathways that were only apparent through the integration of coding and non-coding mutations and remained undetected in the analysis of individual datasets. Thus, our method provides additional information to common approaches that focus on single gene lists.

We evaluated the robustness of ActivePathways to parameter variations and missing data. We varied the parameter *P*_gene_ that determines the ranked gene lists used in the pathway enrichment analysis (default threshold *P*_gene_ < 0.1). The majority of PCAWG cohorts (40/47 or 85%) retrieved significantly enriched pathways even with a considerably more stringent threshold (*P*_gene_ < 0.001), however 67% fewer pathways were found compared to the default threshold in the median cohort (Supplementary Fig. 2). We then evaluated the robustness of ActivePathways to missing data by randomly removing subsets of driver scores from the initial dataset. Even when removing 50% of gene driver *P*-values with *P* < 0.001, the majority of cohorts (37/47 or 79%) had at least one significantly enriched pathway, however 66% fewer pathways were found on average (Supplementary Fig. 3).

We evaluated the expected false positive rates of ActivePathways. We tested 1,000 simulated datasets for each of 47 patient cohorts and expectedly found no significant pathways in 92% of these simulations (Supplementary Fig. 4). Simulated data were obtained by randomly reassigning *P*-values of driver predictions to different genomic elements, a conservative approach that disrupts pathway annotations of genes while retaining the presence of strong *P*-values observed in the real data. The median family-wise false discovery rate of ActivePathways computed across cohorts (7.2%) slightly exceeded the applied multiple testing correction (*Q* < 0.05). Higher rates were observed in cohorts including melanoma tumors, potentially due to abundant promoter mutations caused by impaired nucleotide excision repair in protein-bound genomic regions^44^. We evaluated quantile-quantile (QQ) plots of pathway-based ranked hypergeometric *P*-values from ActivePathways and found that these often deviated from the expected uniform distribution and appeared statistically inflated (Supplementary Fig. 5). However, *P*-values derived from simulated gene scores showed no inflation in our simulations. Anticipating that the most significant cancer driver genes associated with protein-coding mutations, we performed partial simulations. We constructed datasets with simulated gene *P*-values for protein-coding mutations and true *P*-values for non-coding mutations. As expected, partially simulated datasets showed a lesser extent of *P*-value inflation, suggesting that highly significant known cancer genes are responsible for inflation due to their involvement in many pathways. Statistical testing of highly redundant pathways and processes violates the independence assumption of statistical tests and multiple testing procedures, a known caveat of pathway enrichment analysis^1^, which likely explains the observed distribution of significance values of our method. Collectively, these benchmarks show that ActivePathways is a sensitive and robust method for detecting significantly enriched pathways and processes through integrative analysis of multi-omics data.

## Discussion

Integrative pathway enrichment analysis helps distill thousands of high-throughput measurements to a smaller number of pathways and biological themes that are most characteristic of the experimental data at hand, ideally leading to mechanistic insights and candidate genes for follow-up studies. In particular, a joint analysis of complementary datasets often leads to insights that are unavailable in any particular dataset. ActivePathways provides a generally available framework for systematically prioritizing genes and pathways across multiple omics datasets that utilizes fusion of gene significance. This allows us to identify pathways and processes that stand out when the data are combined, yet are not apparent in any single analyzed dataset. The use of ActivePathways is demonstrated in the three case studies above. In our example of cancer driver discovery, pathway analysis provides complementary insights to gene-focused driver discovery as it also focuses on sub-significant genes that are clustered into relevant biological processes and carry coding and non-coding mutations. In the integrative analysis of molecular alterations in breast cancer subtypes, we find a spectrum of genes and pathways whose molecular signatures in the tumors or the microenvironment have potential prognostic significance. A subset of these findings, such as anti-apoptotic signaling, is only apparent through data integration. In the final example, we use ActivePathways to associate the co-expression and DNA-binding networks of Hippo master regulators to downstream pathways and processes with multi-omics evidence.

Our general pathway analysis strategy is applicable to diverse kinds of datasets where well-calibrated *P*-values are available for the entire set of genes or proteins. A multi-omics study may quantify genes using a series of genomic, transcriptomic and proteomic experiments and compute corresponding *P*-values. Data from epigenomic experiments and genome-wide association studies can be also analyzed after signals have been appropriately mapped to genes, for example by identifying ChIP-seq peaks in gene promoters similarly to our GTEx analysis. Clinical and phenotypic information of patients can be also included through association and survival statistics, as shown in our analysis of prognostic signatures of breast cancer subtypes. Our method is expected to work with raw, unadjusted *P*-values and also with *Q*-values adjusted for multiple testing, however it is primarily intended for unadjusted *P*-values for increased sensitivity. ActivePathways conducts multiple testing correction at the pathway level and reports significantly enriched pathways at a family-wise error rate cutoff, regardless of the gene-specific multiple testing correction applied upstream. Quantification of genes and proteins through *P*-values is more robust than quantification through their abundance measures such as counts or fold-changes. *P*-values provided to ActivePathways need to be computed using dedicated methods for individual omics platforms such that inherent biases in the data are accounted for prior to pathway analysis. In our example of cancer driver discovery, appropriately computed *P*-values of driver gene predictions from the PCAWG project^13^ account for confounding factors of somatic mutations, such as gene sequence length and nucleotide content, mutation signatures active in different types of tumors^45^ and biological correlates of mutation frequency such as transcription and replication timing^46^. On the one hand, considering all such variations directly in the pathway enrichment analysis would require substantially more complex models. On the other hand, directly analyzing pathways using simpler metrics (such as mutation counts or frequencies) would propagate any upstream biases to the pathway enrichment analysis and cause challenges with false positives and data interpretation. Thius, given appropriately derived *P*-values for genes, proteins and other molecules, ActivePathways can be applied to a wide range of analyses.

Our method comes with important caveats. First, we only evaluate genes and proteins annotated in pathway databases. Such databases have variable coverage, rely on frequent data updates^47^ and may miss sparsely annotated candidate genes. The most general type of pathway enrichment analysis considers biological processes and molecular pathways however many kinds of gene sets available in resources such as MSigDB^48^ can be used to expand the scope of ActivePathways. Second, pathway information is highly redundant and analyses of rich molecular datasets often result in many significant results reflecting the same underlying pathway. We address this redundancy by visualizing and summarizing pathway results as enrichment maps^1,20^ that summarize multiple similar pathways and processes into general biological themes. Statistical inflation of results accompanied by biological redundancy is addressed by a stringent multiple testing correction. Third, the analysis treats pathways as gene sets and does not consider interactions of genes in pathways. This simplified strategy allows us to consider a wider repertoire of pathways and processes as reliable mechanistic interactions are often context-specific and limited to a small subset of well-studied signaling pathways. Several advanced methods such as HotNet^39^, PARADIGM^49^, and GeneMania^50^ model pathways through gene and protein interactions.

Translation of discoveries into improved human health through actionable mechanistic insights, biomarkers, and molecular therapies is a long-standing goal of biomedical research. For example, next-generation cancer genomics projects such as ICGC-ARGO aim to collect multi-omics datasets with detailed clinical profiles that will present new challenges for pathway and network analysis techniques. In summary, ActivePathways is integrative pathway analysis method that improves systems-level understanding of cellular organization in health and disease.

## Methods

### Integrated and evidence-based gene lists

The main input of ActivePathways is a matrix of *P*-values where rows include all genes of a genome and columns correspond to evidence from omics datasets. To interpret multiple omics datasets, a combined *P*-value is computed for each gene using a data fusion approach, resulting in an integrated gene list. The integrated gene list is computed by merging all *P*-values of a given gene into one combined *P*-value using the Brown’s extension^18^ of the Fisher’s combined probability test that accounts for overall covariation of *P*-values from different sources of evidence. The integrated gene list of Brown *P*-values is then ranked in order of decreasing significance and filtered using a lenient threshold (unadjusted *P* < 0.1 by default). Evidence-based gene lists representing different omics datasets are based on ranked *P*-values from individual columns of the input matrix and filtered using the same significance threshold.

### Statistical enrichment of pathways

Statistical enrichment of pathways in ranked lists of candidate genes is carried out with the ranked hypergeometric test. The test considers one pathway gene set at a time and analyzes increasing subsets of input genes from the top of the ranked gene list. The same procedure is used for integrated and evidence-based gene lists. At each iteration, the test computes the hypergeometric enrichment statistic and *P*-value for the set of genes shared by the pathway and top sub-list of the input gene list. For optimal processing speed, only gene lists ending with a pathway-related gene are considered. The ranked hypergeometric statistic selected the input gene sub-list that achieves the strongest enrichment and the smallest *P*-value as the final result for the given pathway, as:

$$\left( {P_{{{{\mathrm{pathway}}}}},G} \right) = \left\{ {{{{\mathrm{min}}}},{{{\mathrm{argmin}}}}_n} \right\}\mathop {\sum }\limits_{x = k}^{{{{\mathrm{min}}}}\left( {n,K} \right)} \frac{{\left( {\begin{array}{*{20}{c}} K \\ k \end{array}} \right)\left( {\begin{array}{*{20}{c}} {N - K} \\ {n - k} \end{array}} \right)}}{{\left( {\begin{array}{*{20}{c}} N \\ n \end{array}} \right)}},$$

where *P*_pathway_ stands for the hypergeometric *P*-value of the pathway enrichment at the optimal sub-list of the significance-ranked candidate genes, *G* represents the length of the optimal sub-list, i.e., the number of top genes from the input gene list, *N* is the number of protein-coding genes with annotations in the pathway database, i.e., in Gene Ontology and Reactome, *K* is the total number of genes in a given pathway, *n* is the number of genes in a given gene sub-list considered, and *k* is the number of pathway genes in the considered sub-list. For a conservative estimate of pathway enrichment, we consider as background *N* the universe of genes contained in input gene sets (terms from pathway databases and ontologies) rather than the complete repertoire of protein-coding genes. To obtain candidate genes involved in the pathway of interest, we intersect pathway genes with the optimal sub-list of candidate genes. The ranked hypergeometric *P*-value is computed for all pathways and resulting *P*-values are corrected for multiple testing using the Holm-Bonferroni method of family-wise error rate (FWER)^19^. Significant pathways are reported by default (*Q* < 0.05).

### Evaluating omics evidence of enriched pathways

Each evidence-based gene list derived from a single omics dataset is also analyzed for enriched pathways with the ranked hypergeometric test. Pathways found in the integrated gene list are then labeled for supporting evidence in the case they are also found as significant in any evidence-based gene list. A pathway is considered to be found only through data integration and labeled as combined-only if it is identified as enriched in the integrated gene list but not identified as enriched in any of the evidence-based gene lists at equivalent significance cut-offs (default *Q* < 0.05). Each detected pathway is additionally annotated with pathway genes apparent in the optimal sub-list of candidate genes separately for the integrated gene list and each evidence-based gene list.

### Pathways and processes

We used gene sets corresponding to biological processes of Gene Ontology^16^ and molecular pathways of the Reactome database^17^ downloaded from the g:Profiler web server^10^ on Jan 26th 2018. Large general gene sets with more than a thousand genes and small specific gene sets with less than five genes were excluded to avoid statistical inflation of large gene lists and interpretation challenges with very small lists.

### Enrichment map visualization

ActivePathways creates input files for the EnrichmentMap app^20^ of Cytoscape^51^ for network visualization of similar pathways and their coloring according to supporting evidence. Enrichment maps for adenocarcinoma driver mutations, prognostic quantification of molecular alterations of breast cancer, and transcriptional networks downstream of Hippo signaling were visualized with stringent pathway similarity scores (Jaccard and overlap combined coefficient 0.6) and manually curated for the most representative groups of similar pathways and processes. Singleton pathways that were redundant with larger groups of pathways were merged with the latter or discarded. Coloring of pathways in the adenocarcinoma enrichment map was rearranged by merging colors of pathways supported by non-coding mutation scores of promoters, enhancers and/or UTRs into one group.

### Coding and non-coding mutations of the PCAWG dataset

We used ActivePathways to analyze driver predictions of coding and non-coding mutations across white-listed 2583 whole cancer genomes of the ICGC-TCGA PCAWG project. *P*-values of driver predictions were computed separately for protein-coding sequences, promoters, enhancers and untranslated regions (UTR3, UTR5 across multiple subsets of samples representing histological tumor types and pan-cancer cohorts as reported in the PCAWG driver discovery study^13^. We used gene-enhancer mapping predictions provided by PCAWG, excluded enhancers with more than five target genes, and selected the most significant enhancer for each gene, if any. Unadjusted *P*-values for coding sequences, promoters, enhancers and UTRs were compiled as input matrices and analyzed as described above. Missing *P*-values were conservatively interpreted as ones. Results from ActivePathways were validated with two lists of cancer genes. Predicted drivers from the PCAWG consensus analysis^13^ were selected as statistically significant findings (*Q* < 0.05) following a stringent multiple testing correction spanning all types of elements (exons, UTRs, promoter, enhancers). The curated list of known cancer genes was retrieved from the COSMIC Cancer Gene Census (CGC) database^11^. One-tailed Fisher’s exact tests were used to estimate enrichment of these genes in our results, using all human protein-coding genes as the statistical background set.

### Prognostic CNA and mRNA signals in breast cancer

ActivePathways was used to evaluate prognostic pathways in breast cancer subtypes. Multiple types of omics data were used for an integrative analysis: mRNA gene expression data and gene copy number alteration (CNA) data of the were derived from the METABRIC cohort of 1991 patients with a single primary fresh frozen breast cancer specimen each^15^. Curtis et al.^15^ classified the patients into the intrinsic breast cancer subtypes using the PAM50 mRNA-based classifier^52^ resulting in 330 basal-like breast cancers, 238 HER2-enriched breast cancers, 721 luminal-A breast cancers, 491 luminal-B breast cancers. Using these data, we computationally deconvolved tumor cell (TC) mRNA and tumor adjacent cell (TAC) mRNA abundance levels from the bulk profiled specimens. TC mRNA was deconvolved using the ISOpure^21^ method release 2010b in MATLAB. TAC mRNA abundance profiles were computed using the ISOpure.calculate.tac function from the R package ISOpureR^22^ v1.1.2. The deconvolution analyses were performed independently for each breast cancer subtype. The mRNA univariate survival analysis was conducted as follows. For each gene, patients were dichotomized based on mRNA abundance. Dichotomization was either based on the median mRNA abundance for that gene or a fixed value of 6.5. Based on the mRNA abundance distribution of genes on the Y chromosome in female samples, the value 6.5 was estimated as the threshold for noise for non-expressed genes. Median dichotomization was used if the median was above 6.5 or if there were no events in one of the groups when dichotomizing based on 6.5. The high and low groups based on mRNA abundance were compared by univariate log-rank tests for overall patient survival. TC and TAC mRNA abundance values were evaluated independently. Survival modeling was performed in the R statistical environment (v3.4.3) using the survival package (v2.42–3). The CNA univariate survival analysis was conducted as follows. For each gene, we assessed whether more gains or losses were apparent. The copy number status with a higher count was subsequently used to separate patients into two groups: those with the chosen copy number status and the remaining patients. The two groups were then used for overall survival modeling with log-rank tests.

### Hippo pathway target genes in mRNA and ChIP-seq data

This analysis included two types of omics data, mRNA abundance measurements from RNA-seq experiments and transcription factor DNA-binding measurements from chromatin immunoprecipitation sequencing (ChIP-seq) experiments. The RNA-seq dataset of human tissues was downloaded from GTEx v7 data portal (https://www.gtexportal.org/home/). The dataset included transcript abundance values of 21,518 protein-coding genes in 11,688 samples across 53 tissues. Tissues with less than 25 available samples and low gene expression (mean TPM < 1.0; transcripts per million) were excluded from further analysis, resulting in 40 tissues and 9672 samples with mRNA abundance profiles of 19,025 genes. Transcriptional target gene lists for the master transcription factors YAP and TAZ (encoded by *YAP1, WWTR1*) were predicted separately in the following two steps. First, we computed pairwise Pearson correlations between a given TF and all other genes within a tissue of interest and ranked these by the significance *P*-value of positive correlations. Second, the resulting ranked gene lists were then aggregated into one master target gene list across the GTEx tissues using the robust rank aggregation (RRA) method^32^ with default parameters (*Q*_gene_ < 0.05). FDR-adjusted values of genes from RRA for YAP and TAZ were used as the first and second evidence for input of ActivePathways, respectively. For DNA-binding targets of YAP, we retrieved ChIP-seq binding sites in three cell lines (CCLP1, MSTO, and HUCCT1) from an earlier study^33^ that were reprocessed in the ReMap database^34^. Binding sites were filtered by statistical significance (*Q* < 0.05) and mapped to gene promoters of the human genome (hg19) using gene promoters defined in the PCAWG driver analysis^13^. If a promoter had multiple binding sites, the site with the strongest FDR-value was selected as a representative site for that gene. FDR-adjusted values of genes with YAP DNA-binding sites were used as the third evidence for input of ActivePathways. Significantly enriched pathways among the putative target genes of YAP and TAZ were subsequently detected using ActivePathways. We compiled a list of 308 Hippo-related genes from the KEGG pathway database^35^ and two recent review papers^30,31^. To validate the analysis, we tested the overall sets of genes identified by ActivePathways for enrichment of Hippo-related genes using Fisher’s exact tests. The Hippo analysis was conducted separately for two collections of functional gene sets. First, we tested GO biological processes and Reactome pathways similarly to analyses described above. Second, we used gene sets corresponding to transcription factor binding sites (TFBS) derived from the ENCODE project^36^.

### Method benchmarking

We benchmarked ActivePathways using multiple approaches, including simulated datasets of *P*-values, method parameter variations, and partial replacement of *P*-values scores with insignificant *P*-values. Benchmarking was performed on the PCAWG predictions of cancer driver genes with coding and non-coding mutations. To evaluate the false discovery rate of ActivePathways, we created simulated datasets by reassigning all observed *P*-values to random genes and their genomic elements. Simulations were conducted separately for different tumor cohorts. One thousand simulated datasets were analyzed with ActivePathways and those with at least one significantly detected pathway counted towards false discovery rates. A separate set of simulations maintained the positions of non-coding *P*-values among genes and randomly reassigned *P*-values corresponding to protein-coding mutations, expectedly leading to a reduction in detected pathways as the PCAWG datasets primarily included strong *P*-values for genes with frequent protein-coding mutations. Quantile-quantile analysis and QQ-plots were used to compare *P*-value distributions of pathways discovered from true *P*-values, partially shuffled P-values (true non-coding and shuffled protein-coding *P*-values), and fully shuffled *P*-values. To evaluate robustness of ActivePathways, we randomly replaced a fraction of significant driver *P*-values in the true dataset (*P* < 0.001) with insignificant *P*-values (*P* = 1). We tested different fractions of missing values (10, 25, and 50%) across a thousand datasets of randomly selected missing data points. We concluded that most PCAWG cohorts included significantly enriched pathways even with large fractions of missing data. To further evaluate robustness, we tested different values of the Brown *P*-value threshold used to select the integrated gene list for pathway enrichment analysis. The default parameter value (*P*_gene_ < 0.1) was compared to alternative values (0.001, 0.01, 0.05, and 0.2). We concluded that ActivePathways found enriched pathways in most tumor cohorts even at more stringent gene selection levels.

### Ethical review

Sequencing of human subjects’ tissue was performed by ICGC and TCGA consortium members under a series of locally approved Institutional Review Board (IRB) protocols as described in Hudson *et al*. Informed consent was obtained from all human participants. Ethical review of the current data analysis project was granted by the University of Toronto Research Ethics Board (REB) under protocol #30278, “Pan-cancer Analysis of Whole Genomes: PCAWG”.

### Reporting summary

Further information on research design is available in the Nature Research Reporting Summary linked to this article.

## Supplementary information

Supplementary Information

Reporting Summary

## Data Availability

Somatic and germline variant calls, mutational signatures, subclonal reconstructions, transcript abundance, splice calls and other core data generated by the ICGC/TCGA Pan-cancer Analysis of Whole Genomes Consortium is described in the marker paper^4^ and available for download at https://dcc.icgc.org/releases/PCAWG. Additional information on accessing the data, including raw read files, can be found at https://docs.icgc.org/pcawg/data/. In accordance with the data access policies of the ICGC and TCGA projects, most molecular, clinical and specimen data are in an open tier which does not require access approval. To access potentially identifiable information, such as germline alleles and underlying sequencing data, researchers will need to apply to the TCGA Data Access Committee (DAC) via dbGaP (https://dbgap.ncbi.nlm.nih.gov/aa/wga.cgi?page = login) for access to the TCGA portion of the dataset, and to the ICGC Data Access Compliance Office (DACO; http://icgc.org/daco) for the ICGC portion. In addition, to access somatic single nucleotide variants derived from TCGA donors, researchers will also need to obtain dbGaP authorization. Derived PCAWG datasets described specifically in this manuscript can be found at the locations listed below. Additional relevant datasets are listed in the PCAWG study of pathways and networks^14^.

**Table:** 

Label	Synapse ID	ICGC DCC URL	ICGC DCC Filename	Access (Open/Controlled)
PCAWG driver *P*-values	syn8494939	https://dcc.icgc.org/releases/PCAWG/networks/	final_integration_results_2017_03_16.tar.gz	Open
PCAWG pathway and network method results	syn21413360	https://dcc.icgc.org/releases/PCAWG/networks/	pathway_and_network_method_results.tar.gz	Open
PCAWG pathway and network consensus results	syn11654843	https://dcc.icgc.org/releases/PCAWG/networks/	method_results_2017_10_10.tar.gz	Open
Enhancer-gene mappings	syn7201027	https://dcc.icgc.org/releases/PCAWG/networks/	map.enhancer.gene.txt.gz	Open
Coding and non-coding elements	syn21416282	https://dcc.icgc.org/releases/PCAWG/networks/	gene-coding-and-non-coding-elements.tar.gz	Open

## Code availability

ActivePathways is freely available as an R package and source code on the GitHub repository https://github.com/reimandlab/ActivePathways and in the Comprehensive R Archive Network (CRAN). Additional scripts are available upon request. The core computational pipelines used by the PCAWG Consortium for alignment, quality control and variant calling are available to the public at https://dockstore.org/search?search=pcawg under the GNU General Public License v3.0, which allows for reuse and distribution.
