# Supplementary material for: Integrative pathway enrichment analysis of multivariate omics data
Source: Nat Commun. 2020 Feb 5;11:735. doi: 10.1038/s41467-019-13983-9 (PMC7002665; doi:10.1038/s41467-019-13983-9)
Supplement: Supplementary file 2 — Reporting Summary [file 41467_2019_13983_MOESM2_ESM.pdf]

nature research

Corresponding author(s): Jüri Reimand

Last updated by author(s): Jul 6, 2019

Reporting Summary

Nature Research wishes to improve the reproducibility of the work that we publish. This form provides structure for consistency and transparency in reporting. For further information on Nature Research policies, see Authors & Referees and the Editorial Policy Checklist.

Statistics

For all statistical analyses, confirm that the following items are present in the figure legend, table legend, main text, or Methods section.

n/a	Confirmed	
☐	☒	The exact sample size (n) for each experimental group/condition, given as a discrete number and unit of measurement
☒	☐	A statement on whether measurements were taken from distinct samples or whether the same sample was measured repeatedly
☐	☒	The statistical test(s) used AND whether they are one- or two-sided<br>*Only common tests should be described solely by name; describe more complex techniques in the Methods section.*
☒	☐	A description of all covariates tested
☐	☒	A description of any assumptions or corrections, such as tests of normality and adjustment for multiple comparisons
☒	☐	A full description of the statistical parameters including central tendency (e.g. means) or other basic estimates (e.g. regression coefficient) AND variation (e.g. standard deviation) or associated estimates of uncertainty (e.g. confidence intervals)
☒	☐	For null hypothesis testing, the test statistic (e.g. F, t, r) with confidence intervals, effect sizes, degrees of freedom and P value noted<br>*Give P values as exact values whenever suitable.*
☒	☐	For Bayesian analysis, information on the choice of priors and Markov chain Monte Carlo settings
☒	☐	For hierarchical and complex designs, identification of the appropriate level for tests and full reporting of outcomes
☐	☒	Estimates of effect sizes (e.g. Cohen's d, Pearson's r), indicating how they were calculated

Our web collection on statistics for biologists contains articles on many of the points above.

Software and code

Policy information about availability of computer code

Data collection	No software was used for data collection.
Data analysis	ActivePathways is freely available as an R package and source code on the GitHub repository https://github.com/reimandlab/ActivePathways and in the Comprehensive R Archive Network (CRAN). Additional scripts are available upon request.

For manuscripts utilizing custom algorithms or software that are central to the research but not yet described in published literature, software must be made available to editors/reviewers. We strongly encourage code deposition in a community repository (e.g. GitHub). See the Nature Research guidelines for submitting code & software for further information.

Data

Policy information about availability of data

All manuscripts must include a data availability statement. This statement should provide the following information, where applicable:

- Accession codes, unique identifiers, or web links for publicly available datasets
- A list of figures that have associated raw data
- A description of any restrictions on data availability

The PCAWG data of simple somatic mutations, coding and non-coding cancer driver calls, RNA expression values, copy number somatic mutations and structural somatic mutations are available at the ICGC DCC (https://dcc.icgc.org/pcawg).

Field-specific reporting

Please select the one below that is the best fit for your research. If you are not sure, read the appropriate sections before making your selection.

☒ Life sciences ☐ Behavioural & social sciences ☐ Ecological, evolutionary & environmental sciences

For a reference copy of the document with all sections, see nature.com/documents/nr-reporting-summary-flat.pdf

Life sciences study design

All studies must disclose on these points even when the disclosure is negative.

Sample size	No sample-size calculations were used. Pathway analyses were performed on gene-based p-values derived from all samples in PCAWG and METABRIC.
Data exclusions	In the GTEx analysis we excluded tissue types where few samples with available transcriptomics data were available.
Replication	N/A
Randomization	N/A
Blinding	N/A

Reporting for specific materials, systems and methods

We require information from authors about some types of materials, experimental systems and methods used in many studies. Here, indicate whether each material, system or method listed is relevant to your study. If you are not sure if a list item applies to your research, read the appropriate section before selecting a response.

Materials & experimental systems

n/a	Involved in the study	
☒	☐	Antibodies
☒	☐	Eukaryotic cell lines
☒	☐	Palaeontology
☒	☐	Animals and other organisms
☐	☒	Human research participants
☒	☐	Clinical data

Methods

n/a	Involved in the study	
☒	☐	ChIP-seq
☒	☐	Flow cytometry
☒	☐	MRI-based neuroimaging

Human research participants

Policy information about studies involving human research participants

Population characteristics	Sequencing of human subjects' tissue was performed by ICGC and TCGA consortium members under a series of locally approved Institutional Review Board (IRB) protocols as described in Hudson et al.
Recruitment	Informed consent was obtained from all human participants.
Ethics oversight	Ethical review of the current data analysis project was granted by the University of Toronto Research Ethics Board (REB) under protocol #30278, "Pan-cancer Analysis of Whole Genomes: PCAWG".

Note that full information on the approval of the study protocol must also be provided in the manuscript.
